# Geochemistry and Microtextures of Vein Calcites Pervading the Izu‐Bonin Forearc and Rear Arc Crust: New Insights From IODP Expeditions 352 and 351

**DOI:** 10.1029/2019GC008745

**Published:** 2020-02-19

**Authors:** D. Quandt, P. Micheuz, W. Kurz, S. M. Bernasconi, D. Hippler, K. Krenn, C. A. Hauzenberger

**Affiliations:** ^1^ NAWI Graz Geocenter, Institute of Earth Sciences University of Graz Austria; ^2^ Department of Earth Sciences, Geological Institute ETH Zurich Zurich Switzerland; ^3^ NAWI Graz Geocenter, Institute of Applied Geosciences Graz University of Technology Austria

**Keywords:** Izu‐Bonin forearc and rear arc, Calcite veins, Geochemistry, IODP 352 and 351, Microtextures

## Abstract

International Ocean Discovery Program Expeditions 352 and 351 drilled into the Western Pacific Izu‐Bonin forearc and rear arc. The drill cores revealed that the forearc is composed of forearc basalts (FAB) and boninites and the rear arc consists of FAB‐like rocks. These rocks are pervaded by calcite veins. Blocky vein microtextures enclosing host rock fragments dominate in all locations and suggest hydrofracturing and advective fluid flow. Significant diffusion‐fed and crystallization pressure‐driven antitaxial veining is restricted to the rear arc. The lack of faults and presence of an Eocene sedimentary cover in the rear arc facilitated antitaxial veining. Rare earth element and isotopic (δ^18^O, δ^13^C, ^87^Sr/^86^Sr, and Δ_47_) tracers indicate varying parental fluid compositions ranging from pristine to variably modified seawater. The most pristine seawater signatures are recorded by FAB‐hosted low‐T (<30 °C) vein calcites. Their ^87^Sr/^86^Sr ratios intersect the ^87^Sr/^86^Sr seawater curve at ~35–33 and ~22 Ma. These intersections are interpreted as precipitation ages, which concur with Pacific slab rollback. Boninite‐hosted low‐T (<30 °C) vein calcites precipitated from seawater that was modified by fluid‐rock interactions. Mixing calculations yield a mixture of >95% seawater and <5% basaltic ^87^Sr/^86^Sr. In the rear arc, low‐T rock alteration lowered the circulating seawater in δ^18^O and ^87^Sr/^86^Sr. Thus, vein calcites precipitated from modified seawater with up to 20–30% basaltic ^87^Sr/^86^Sr at temperatures up to 74 ± 12 °C. These results show how the local geology and vein growth dynamics affect microtextures and geochemical compositions of vein precipitates.

## Key Points

- In the Izu‐Bonin forearc vein calcites precipitated at <30 °C from pristine to weakly modified seawater in hydrofractures
- Calcite veining (max. 74 °C) in the rear arc is characterized by weak to moderate fluid‐rock interaction, diffusive and advective fluid flow
- A sedimentary cover and the lack of faults facilitated diffusion‐fed and crystallization pressure‐driven antitaxial veining in the rear arc

## 1 Introduction

**Figure 1:**
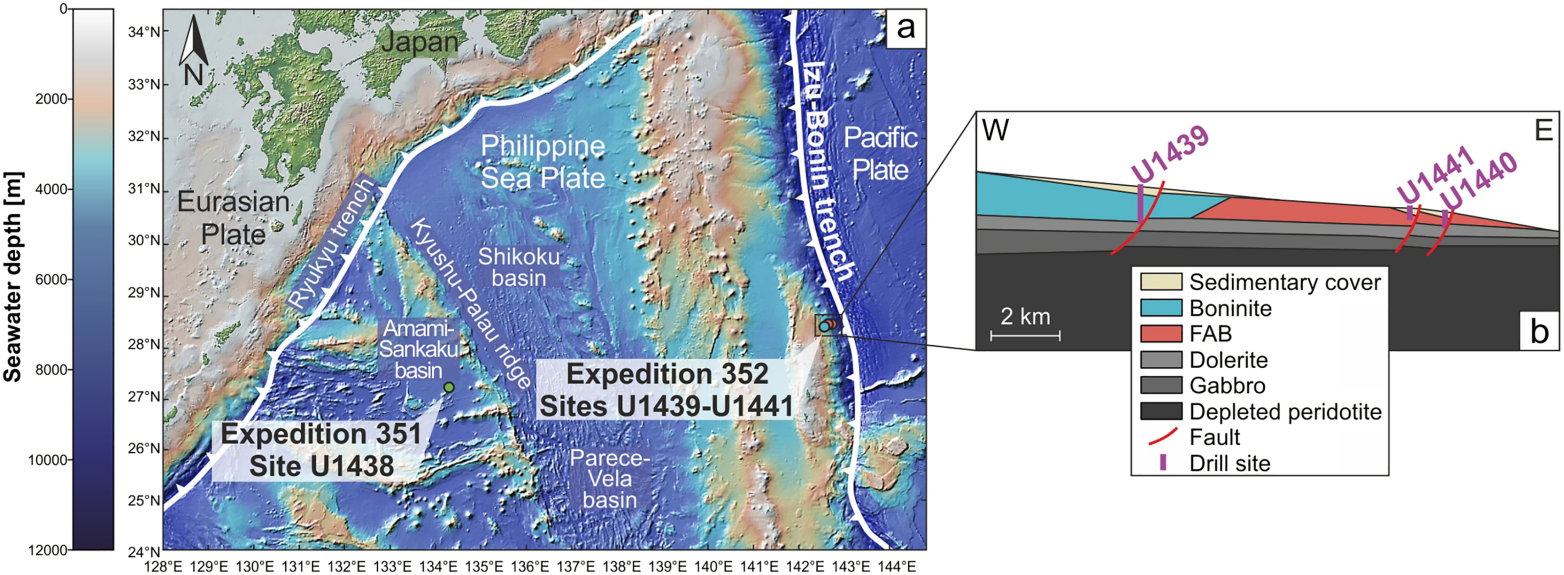
(a) Map of the Western Pacific highlighting the IODP Expedition 352 Sites U1439–U1441 located in the Izu‐Bonin forearc (Reagan et al., 2015) and IODP Expedition 351 Site U1438 situated in the rear arc (Amami‐Sankaku basin) (Arculus, Ishizuka, Bogus, & the Expedition 351 Scientists, 2015). (b) Schematic E‐W cross section through the Izu‐Bonin forearc indicating the lithological succession and drill sites (Reagan et al., 2015).

The knowledge of the stratigraphy, structure, and geochemical composition of the oceanic crust is based on ophiolites and drill cores from the International Ocean Discovery Program (IODP) and its predecessors. Since ophiolites mostly experienced a complex history of uplift and emplacement on continental crust, their structures and geochemical compositions may have been overprinted and/or altered. In situ drilling through oceanic crust can provide more pristine rocks and structures that have not been involved in uplift and emplacement processes. With the aim of investigating the tectonomagmatic processes of subduction initiation, IODP Expedition 352 drilled into the Izu‐Bonin forearc crust in the Western Pacific (Figure 1) and successfully cored 1.22 km of volcanic rocks (Reagan et al., 2015). The four drill sites revealed two well‐preserved geochemically distinct volcanic rock sequences–forearc basalts (FAB) and boninites–each related to different stages after subduction initiation of the Pacific Plate beneath the Philippine Sea Plate in the early Eocene (Reagan et al., 2017; Reagan et al., 2019). Drill cores that were recovered during the preceding IODP Expedition 351 (Figure 1) unveiled that the Izu‐Bonin rear arc crust is composed of FAB‐like rocks but lacks boninites (Arculus, Ishizuka, Bogus, Gurnis, et al., 2015; Arculus, Ishizuka, Bogus, & the Expedition 351 Scientists, 2015; Hickey‐Vargas et al., 2018; Ishizuka et al., 2018).

The volcanic units of the Izu‐Bonin forearc and rear arc are substantially and similarly pervaded by veins that document extensive fluid flow through the oceanic crust and subsequent secondary mineral precipitation (Arculus, Ishizuka, Bogus, & the Expedition 351 Scientists, 2015; Christeson et al., 2016; Reagan et al., 2015). Numerous previous studies on calcite veins pervading the oceanic crust used δ^18^O, δ^13^C, and ^87^Sr/^86^Sr isotopic compositions, rare earth element and yttrium (REE+Y) concentrations, and fluid inclusions to investigate the geochemical composition of circulating fluids, and the age and temperature of secondary mineral precipitation (e.g., Alt & Teagle, 2003; Alt et al., 1998; Brandstätter et al., 2016; Brandstätter et al., 2018; Hart & Staudigel, 1978; Quandt et al., 2018; Quandt, Micheuz, Kurz, Kluge, et al., 2019; Schroeder et al., 2015). However, studies on calcite veins from the Izu‐Bonin‐Mariana trench in particular are rare. They are either confined to stable isotopic data (Alt et al., 1998) or focus on processes in the Mariana subduction channel at greater depth (Albers et al., 2019). Most of these geochemical studies on vein calcites from the Izu‐Bonin‐Mariana trench and other sections of oceanic crust including ophiolites have in common that they do not consider vein microtextures. Previous structural studies classified veins into different types indicative of the mode of fracturing, fluid flow, and fracture sealing (Bons et al., 2012, and references therein); investigated the kinematic histories of veins and their host rocks (e.g., Brandstätter et al., 2017; Durney & Ramsay, 1973; Ramsay & Huber, 1983); or simulated veining in numerical models and laboratory experiments (e.g., Bons, 2001; Hilgers et al., 2004; Hilgers et al., 2001; Hilgers & Urai, 2002; Means & Li, 2001; Nollet et al., 2005; Taber, 1916) without considering geochemical data.

Here we present a multidisciplinary study on vein calcites pervading the volcanic units of the Izu‐Bonin forearc (IODP Expedition 352) and rear arc (IODP Expedition 351), which links geochemical with microtextural data. This study (1) investigates the physicochemical conditions of fluid flow and vein calcite formation, (2) evaluates the extent of fluid‐rock interaction, (3) constrains the precipitation ages of seawater‐derived vein calcites, (4) examines if the different calcite vein types correlate with specific geochemical signatures, and (5) compares the postmagmatic evolution of the Izu‐Bonin forearc and rear arc. For this purpose, different geochemical tracers and proxies are used, and the local geological characteristics of the forearc and rear arc are considered. REE+Y concentrations, as well as radiogenic strontium (^87^Sr/^86^Sr), stable oxygen (δ^18^O) and carbon (δ^13^C), and clumped (Δ_47_) isotopic compositions record the geochemical compositions of circulating fluids and temperature of vein mineral precipitation. The new clumped isotope method (Ghosh et al., 2006) has been rarely applied on secondary calcites from the oceanic crust (Coogan et al., 2019; Quandt, Micheuz, Kurz, Kluge, et al., 2019). In contrast to δ^18^O thermometric calculations, clumped isotopes yield precipitation temperatures without requiring knowledge of the parental δ^18^O fluid composition, which in turn may be calculated (Ghosh et al., 2006). Intersections of ^87^Sr/^86^Sr ratios of purely seawater‐derived vein calcites with the well‐established Sr isotope seawater curve (McArthur et al., 2001) give estimates on the time of calcite precipitation (e.g., Hart & Staudigel, 1978). These geochemical tracers are combined with Raman spectroscopy, and thin section and cathodoluminescence (CL) petrography, which provide insights into the vein mineralogy and modes of fracturing, fluid flow, and fracture sealing. This combination of geochemical and microtextural data improves the understanding of vein formation in the oceanic crust.

## 2 Geological Background

### 2.1 Geological Setting of the Izu‐Bonin Forearc and Rear Arc

**Figure 2:**
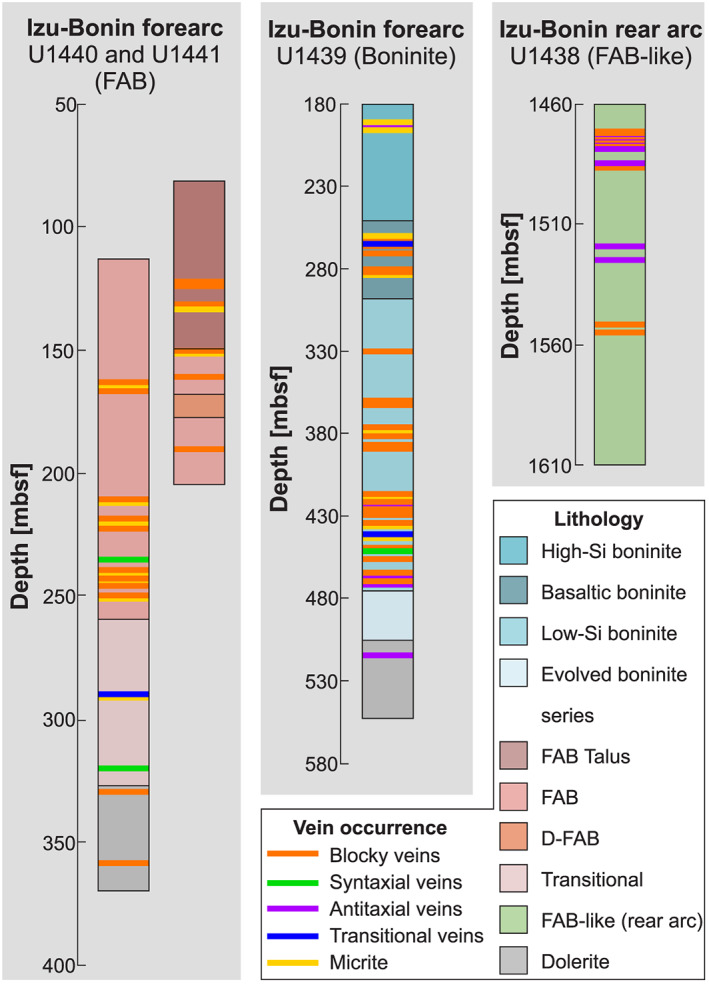
Stratigraphy at Sites U1438–U1441 (Arculus, Ishizuka, Bogus, Gurnis, et al., 2015; Arculus, Ishizuka, Bogus, & the Expedition 351 Scientists, 2015; Reagan et al., 2015; Reagan et al., 2017; Reagan et al., 2019) and occurrence of vein types with depth in meters below seafloor (mbsf).

The prominent seafloor topography of the Izu‐Bonin trench in the Western Pacific reflects the steep subduction of the Pacific Plate under the Philippine Sea Plate over a distance of 3,000 km (e.g., Bloomer et al., 1995; Faccenna et al., 2018; Holt et al., 2018; Stern & Bloomer, 1992). In order to better understand the initiation of this intraoceanic subduction zone, IODP Expedition 352 penetrated the volcano‐stratigraphic succession of the Izu‐Bonin forearc crust (Reagan et al., 2015). Four sites revealed FAB (Sites U1440 and U1441) and boninites (Sites U1439 and U1442), which are stratigraphically and geochemically distinguishable (Figure 2) (Reagan et al., 2015; Reagan et al., 2017; Reagan et al., 2019). Each volcanic rock sequence formed as a response to the consecutive stages of subduction initiation and represents a modern analog to many suprasubduction zone ophiolites (Reagan et al., 2017; Reagan et al., 2019).

Magmatic activity in the Izu‐Bonin protoforearc initiated ~52 Ma when the dense Pacific Plate subsided into the mantle along a transform fault or fracture zone and induced asthenospheric upwelling (Ishizuka, Tani, et al., 2011; Ishizuka et al., 2018; Reagan et al., 2013; Reagan et al., 2019). This early subduction initiation stage caused decompression melting that generated mainly aphyric basaltic pillow lavas, sheet flows, and hyaloclastites with FAB composition (Reagan et al., 2017; Reagan et al., 2019; Shervais et al., 2019). FAB show a similar major element geochemistry (MgO, FeO*, and CaO) as mid‐ocean ridge basalts but have lower Zr/Y and Ti/V ratios (Reagan et al., 2017; Shervais et al., 2019). As subduction of the Pacific Plate continued, fluid‐induced melting became dominant over decompression melting within ~2 Myr after subduction initiation and generated vesicle‐rich porphyritic pillow lavas and massive sheet flows of boninitic composition (Ishizuka, Tani, et al., 2011; Reagan et al., 2017; Reagan et al., 2019). Boninites are defined as high‐MgO (>8 wt.%) and low‐TiO_2_ (<0.5 wt.%) volcanic rocks with >52 wt.% SiO_2_ (Le Bas, 2000). In the Izu‐Bonin forearc they have been subdivided into low‐Si and high‐Si varieties (Reagan et al., 2017).

IODP Expedition 351 drilled through the Izu‐Bonin rear arc crust in the Amami‐Sankaku basin (Site U1438), which separated from the forearc after rifting of the Kyushu‐Palau ridge and spreading in the Shikoku and Parece‐Vela basins between ~29 and ~15 Ma (Arculus, Ishizuka, Bogus, Gurnis, et al., 2015; Arculus, Ishizuka, Bogus, & the Expedition 351 Scientists, 2015; Okino et al., 1998; Sdrolias et al., 2004). Drill cores revealed that the rear arc crust is composed of lava sheet flows of FAB‐like composition, whereas boninites are absent (Figure 2). These FAB‐like rocks are associated with the earliest stage of subduction initiation and formed between ~49 and ~47 Ma (Brandl et al., 2017; Hickey‐Vargas et al., 2018; Ishizuka et al., 2018).

The postmagmatic phase of the Izu‐Bonin forearc is characterized by graben and half‐graben formation along conjugate normal faults that acted as prominent fluid paths along which secondary minerals precipitated in veins (Christeson et al., 2016; Kurz et al., 2019; Reagan et al., 2015). These structures are late‐stage features that concur with Eocene‐Miocene Pacific slab rollback (Faccenna et al., 2018; Kurz et al., 2019; Robertson et al., 2018). Drill cores and seismic cross sections from the rear arc in contrast lack any major fault structures (Arculus, Ishizuka, Bogus, Gurnis, et al., 2015; Arculus, Ishizuka, Bogus, & the Expedition 351 Scientists, 2015). Deposition of pelagic carbonates on the volcanic forearc units began ~35 Ma (Robertson et al., 2018). This implies an up to ~15 Myr long hiatus after basaltic volcanism due to topographic isolation or bypassing sediment (Robertson et al., 2018). In contrast, the rear arc basement is overlain by mudstones and sandstones, which were deposited during volcanic activity (Arculus, Ishizuka, Bogus, Gurnis, et al., 2015; Ishizuka et al., 2018). These sediments show volcaniclastic inputs from adjacent arc edifices (Arculus, Ishizuka, Bogus, & the Expedition 351 Scientists, 2015; Brandl et al., 2017).

### 2.2 Vein Macrostructures and Host Rock Alteration

Macroscale vein structures and host rock alteration characteristics were described by Arculus, Ishizuka, Bogus, & the Expedition 351 Scientists (2015), Reagan et al. (2015), and Hickey‐Vargas et al. (2018) and are summarized here. The drill cores revealed that the Izu‐Bonin forearc and rear arc sites are pervaded by vertical to subvertical (mostly >45°) veins at different depths. Veins document mineralized (1) (tapered) tension fractures; (2) hybrid (tension and shear) fractures; (3) tension gashes linked to releasing bends and extensional step‐overs, which are arranged along shear fractures; and (4) extensional horsetail splay faults emanating from single sets of shear fractures with top‐down displacement (normal sense of shear). These structures occur independently of the drill site and depth (Figure 2) and may be conjugated, constituting complex vein networks. The typical proportion of veins within a rock volume is <10% but may increase up to ~75% where fault‐related breccias are cemented.

The extent of host rock alteration varies and depends on drill site and lithology. In general, microcrystalline groundmasses, olivine, orthopyroxene, and plagioclase phenocrystals may be replaced by smectite group clay minerals, zeolites, and calcite. Glass may be fresh, devitrified, or altered to clay minerals, zeolites, and palagonite. Chlorite is an additional alteration product in FAB‐like rocks at Site U1438 where alteration is localized and variable. At Site U1439 the degree of alteration of boninites increases with depth. Where host rocks are intensely brecciated, pervasive alteration resulted in significantly modified geochemical host rock compositions. FAB at Site U1440 are comparatively fresh. More intense alteration is restricted to the transition zone and dolerite dykes. At Site U1441 FAB alteration is variable and decreases with depth. In contrast to boninites, FAB and FAB‐like rocks show argillaceous vein selvedges and vein‐parallel alteration halos. Hence, alteration in FAB and FAB‐like rocks tends to be more localized, whereas boninites were rather pervasively altered.

## 3 Methods

### 3.1 Sample Preparation

Raman spectroscopy and CL petrography were performed on polished thin sections. Rock slices used for thin section preparation were subsequently used for laser ablation analyses. For stable, radiogenic Sr, and clumped isotope analyses, fine‐grained powders of vein calcites were obtained using a computer‐controlled (*x*‐*y*‐*z* position) micromill device (ESI, Portland) with 100 μm diameter (equipped with a digital camera) or a handheld dentist drill at the NAWI Graz Geocenter, Institute of Earth Sciences, University of Graz (Austria). Fine‐grained powders necessary for all isotope analyses were produced by separate drilling into vein calcites hosted in rock slices. Except for a few reported samples, this means that every analysis is based on a different sample powder even if two analyses belong to the same sample. This enabled sampling of different calcite generations and vein branches from the same sample. It also facilitated the detection of potential isotopic heterogeneities. The low thickness of some veins and large amount of powder necessary for Sr isotopic analyses required separate drilling spots in order to obtain sufficient sample powder and to avoid contamination with host rock material.

### 3.2 Raman Spectroscopy

Confocal Raman spectra indicative of vein mineral phases were obtained with a HORIBA Jobin Yvon LabRam‐HR 800 Raman microspectrometer at the NAWI Graz Geocenter, Institute of Earth Sciences, University of Graz (Austria). Crystals within polished thin sections were excited at room temperature with the 532 nm emission line of a 100 mW Nd‐YAG laser through an OLYMPUS 100X objective (numerical aperture 0.9). The laser spot on the surface had a diameter of approximately 2 μm and a power of about 20 mW. Light was dispersed by a holographic grating with 1,800 grooves/mm. The slit width was set to 100 μm. The dispersed light was collected by a 1,024 × 256 nitrogen‐cooled, open‐electrode charge‐coupled device detector. Spectra were recorded unpolarized. Band shifts were calibrated by regularly adjusting the zero position of the grating and controlled by measuring the Rayleigh line of the incident laser beam. The detection range of the Raman shift lies between 100 and 1,200 cm^−1^ using an acquisition time between 10 and 20 s.

### 3.3 Cathodoluminescence

CL of polished and carbon‐coated thin sections was analyzed using a hot cathode CL microscope (Lumic HC5‐LM) at the NAWI Graz Geocenter, Institute of Earth Sciences, University of Graz (Austria). A voltage of 13–14 kV accelerated electrons under a vacuum of <10^−5^ mbar and produced true‐color CL in the visible spectrum. CL photographs were acquired in real time using an attached digital camera.

### 3.4 Stable Oxygen and Carbon Isotopes

A set of 38 representative samples was chosen for stable oxygen and carbon isotope analyses. After the reaction of the sample powders with oversaturated 100% orthophosphoric acid at 70 °C in a Kiel II automated reaction system, stable oxygen and carbon isotopes were measured using a Delta^Plus^ isotope ratio mass spectrometer at the NAWI Graz Geocenter, Institute of Earth Sciences, University of Graz (Austria). The powders of further 22 samples were reacted with phosphoric acid at 75 °C and subsequently measured by continuous‐flow isotope ratio mass spectrometry using a GasBench II coupled to a Finnigan DELTAplus XP mass spectrometer at the JR‐AquaConSol GmbH, Graz (Austria). The reproducibility of replicate analyses for standards (in‐house and NBS 19) and samples of both measurements was better than ±0.15‰ Vienna Pee Dee Belemnite (VPDB) for δ^18^O and ±0.1‰ VPDB for δ^13^C.

Precipitation temperatures were calculated assuming equilibrium precipitation from a parental fluid with a δ^18^O value of −1‰ Vienna Standard Mean Ocean Water (VSMOW) (e.g., Epstein et al., 1953; McCrea, 1950; Urey, 1947) corresponding to ice‐free Eocene seawater (Burgess et al., 2008; Zachos et al., 2001) and using the calcite‐water fractionation curve of Friedman and O'Neil (1977), which is valid for temperatures between 0 and 500 °C. Additional parental fluid δ^18^O values of 0 and +1‰ VSMOW were also applied in order to consider potential ^18^O depletion or enrichment due to fluid‐rock interactions or fluid mixing.

### 3.5 Clumped Isotopes

The clumped isotope compositions of vein calcites were determined at the Department of Earth Sciences, Geological Institute, ETH Zurich (Switzerland) using a Thermo Fisher Scientific 253Plus mass spectrometer coupled to a Kiel IV carbonate preparation device, following the method described in Schmid and Bernasconi (2010), Meckler et al. (2014), and Müller et al. (2017). The Kiel IV device included a PoraPakQ trap to eliminate potential organic contaminants. Prior to each sample run, the pressure‐dependent backgrounds were determined on all beams to correct for nonlinearity effects in the mass spectrometer according to Bernasconi et al. (2013). During each run, 18 replicates of 130–150 μg of different samples and 6 replicates of each of the four carbonate standards, ETH‐1, ETH‐2, ETH‐3, and ETH‐4 (Bernasconi et al., 2018), were analyzed. Each analysis consisted of 18 cycles of sample and reference gas comparison, with 26 seconds integration. The calculations and corrections were done with the software Easotope (John & Bowen, 2016) using the revised “Brand parameters” for ^17^O correction as suggested by Daëron et al. (2016). Individual sample replicate and standard measurements are archived at PANGAEA Data Archiving and Publication (Quandt, Micheuz, Kurz, Bernasconi, et al., 2019).

Temperatures were calculated using the Kele et al. (2015) calibration recalculated with the “Brand parameters” and the new accepted values for the ETH standards as reported in Bernasconi et al. (2018). We use this calibration because it has been produced with the same instrumentation and data reduction schemes as the data presented in this study and thus guarantees robust temperature estimates avoiding uncertainties related to poor interlaboratory comparability (Petersen et al., 2019).

In contrast to the δ^18^O geothermometer, the clumped isotope method yields temperature estimates without the necessity to know the parental fluid δ^18^O value (e.g., Ghosh et al., 2006). Conversely, this parental fluid δ^18^O value was calculated based on the clumped isotope temperature using the calibrations of Coplen (2007) and Kim and O'Neil (1997). Both calibrations result in a range of parental fluid δ^18^O values for every sample, which reflects the ongoing debate on the isotopic fractionation factor.

A theoretically long heating rate of ≥90–100 °C over tens of Myr is necessary to reorder the ^18^O‐^13^C bonds of calcite (Passey & Henkes, 2012; Stolper & Eiler, 2015). Because of this long duration compared to the relatively short phase of magmatic activity (~2 Myr) in the Izu‐Bonin forearc (Ishizuka, Tani, et al., 2011; Reagan et al., 2013; Reagan et al., 2019), we suggest that the clumped isotope temperature and thus calculated parental fluid δ^18^O values have not been affected by reordering.

### 3.6 Radiogenic Strontium Isotopes

Prior to Sr separation via ion exchange column chemistry, 15–30 mg powder per sample were dissolved in double‐distilled 0.4 M HCl in order to avoid dissolution of noncarbonate vein or host rock constituents. Subsequently, Sr separation was performed via ion exchange column chemistry using Sr‐specific extraction chromatographic resin (Eichrom®, USA) and diluted double‐distilled 3 M HNO_3_ for column conditioning. Five milliliter of 3 M HNO_3_ and 1 ml of 0.1 M HNO_3_ were used for element elution and Sr collection, respectively (for details, see Stammeier et al., 2018).

Strontium isotope measurements were carried out in wet‐plasma mode using a Nu Plasma II multicollector inductively coupled plasma mass spectrometer (Nu instruments, Wrexham, UK) equipped with a 0.1 ml/min MicroMist® glass nebulizer and an ASX‐110FR Flowing Rinse MicroAutosampler (Teledyne‐Cetac Technologies) at the NAWI Graz Geocenter, Central Lab for Water, Minerals and Rocks, Graz University of Technology (Austria). In summary, samples were measured (1) with sensitivities for ^88^Sr being generally better than 20 V per 500 μg/ml, (2) as one block of 25 cycles with an integration time of 5 s, and (3) with background determined on half‐masses prior to each block measured. Measured isotope ratios were internally normalized to an ^86^Sr/^88^Sr ratio of 0.1194. The analytical uncertainty of the ^87^Sr/^86^Sr measurement is ±0.00001, and repeated analysis of NIST NBS 987 (National Institute of Standards and Technology, Gaithersburg, USA) within the analytical session gave ^87^Sr/^86^Sr ratios of 0.710253 ± 0.000067 (2 SD; *n* = 12). Total procedural blanks below 1.2 ng Sr are negligible compared to analyte signals.

The decay of ^87^Rb to ^87^Sr affects the ^87^Sr/^86^Sr sample ratio and may require the calculation of the initial ^87^Sr/^86^Sr ratio at the time of sample formation. However, Rb is usually not incorporated into the calcite lattice (McArthur et al., 2012) and analyzed Rb concentrations are <1 ppm. In addition, the decay rate of Rb is low compared to the maximum calcite formation ages, which are constrained by the emplacement of host rocks in Eocene times. Therefore, calculation of initial ^87^Sr/^86^Sr ratios at the time of calcite formation is not required.

### 3.7 Rare Earth Elements and Yttrium

Prior to the analysis, rock slices containing calcite veins were polished and cleaned. REE+Y concentrations of vein calcites were determined using a 193 nm New Wave ArF excimer laser coupled to an Agilent 7500cx inductively coupled plasma mass spectrometer (LA‐ICP‐MS) at the NAWI Graz Geocenter, Central Lab for Water, Minerals and Rocks, Graz University of Technology (Austria). At a frequency of 7 Hz, the laser ablated between 6 and 20 spots per sample. Each laser spot was set to 75 μm diameter and placed along profiles through the vein samples. The acquisition time was set to a 30 s gas blank followed by a 60 s dwell time for each spot. The NIST standard reference material SRM 612 (National Institute of Standards and Technology, Gaithersburg, USA) was used to standardize the LA‐ICP‐MS analyses (Jochum et al., 2012, and references therein) before and after a block of 20 sample spot measurements. SRM 610 and 614 (National Institute of Standards and Technology, Gaithersburg, USA) and synthetic carbonate standard MACS‐3 (U.S. Geological Survey) were analyzed as unknowns to monitor accuracy. All reference materials were reproduced for most elements within errors for the used elements. The relative uncertainty of REE+Y analyses of the NIST SRM 612 standard measurements was <5%. Time‐resolved LA‐ICP‐MS data reduction was carried out using the GLITTER 4.0 software (Macquarie University, Sydney). The element concentrations are archived at PANGAEA Data Archiving and Publication (Quandt, Micheuz, Kurz, Bernasconi, et al., 2019).

In order to detect potential contamination of vein calcites during LA‐ICP‐MS analyses due to the ablation of clay minerals and/or zeolites, thresholds of Al and Zr concentrations were applied. Al concentrations <2000 ppm and Zr concentrations <5 ppm are interpreted as uncontaminated calcite analyses (Schier et al., 2018). Most analyzed samples have Zr and Al concentrations below the respective thresholds. Only 10 out of 135 laser ablation spot analyses reveal Al concentrations slightly above the Al threshold (Al <8,400 ppm). Most spot analyses with Al >2,000 ppm show similar REE+Y distribution patterns as spot analyses with Al <2000 ppm from the same sample. Therefore, these analyses are considered as representative and were not rejected.

REE+Y concentrations were normalized to the Post‐Archean Australian Shale (PAAS) values from McLennan (1989). For the calculation of PAAS‐normalized Ce anomalies (Ce/Ce*_PAAS_ = Ce_PAAS_/[Pr_PAAS_^2^/Nd_PAAS_]), the potential anomalous behavior of La (Lawrence et al., 2006; Tostevin et al., 2016) was considered. PAAS‐normalized Eu anomalies were calculated using the neighboring REEs (Eu/Eu*_PAAS_ = 2 × Eu_PAAS_/[Sm_PAAS_+Gd_PAAS_]) (Tostevin et al., 2016). Seemingly positive Eu anomalies may be caused by analytical interferences due to elevated Ba concentrations and inherited from shale normalization values (Tostevin et al., 2016). Low average Ba concentrations <0.5 ppm and average Ba/Eu ratios <15 argue against analytical interferences. However, PAAS‐normalized Eu anomalies are 30% larger than chondrite‐normalized Eu anomalies (McDonough & Sun, 1995). Therefore, only PAAS‐normalized Eu anomalies >1.3 are considered as true positive anomalies.

## 4 Results

### 4.1 Vein Mineralogy

Calcite is the dominant vein mineral throughout the drill cores from the Izu‐Bonin forearc and rear arc. Other common mineralogical phases include (1) euhedral zeolites that typically occur as early‐stage precipitates preceding calcite, (2) late‐stage zeolite veins crosscutting calcite veins, (3) accessory palagonite and opaque minerals associated with calcite, (4) narrow argillaceous vein selvedges occasionally accompanied by alteration halos, and (5) host rock solid inclusion bands and trails. In the Izu‐Bonin rear arc, zeolites are completely absent. The only noncarbonate phases in rear arc veins are argillaceous vein selvedges and host rock bands, trails, and fragments.

**Figure 3:**
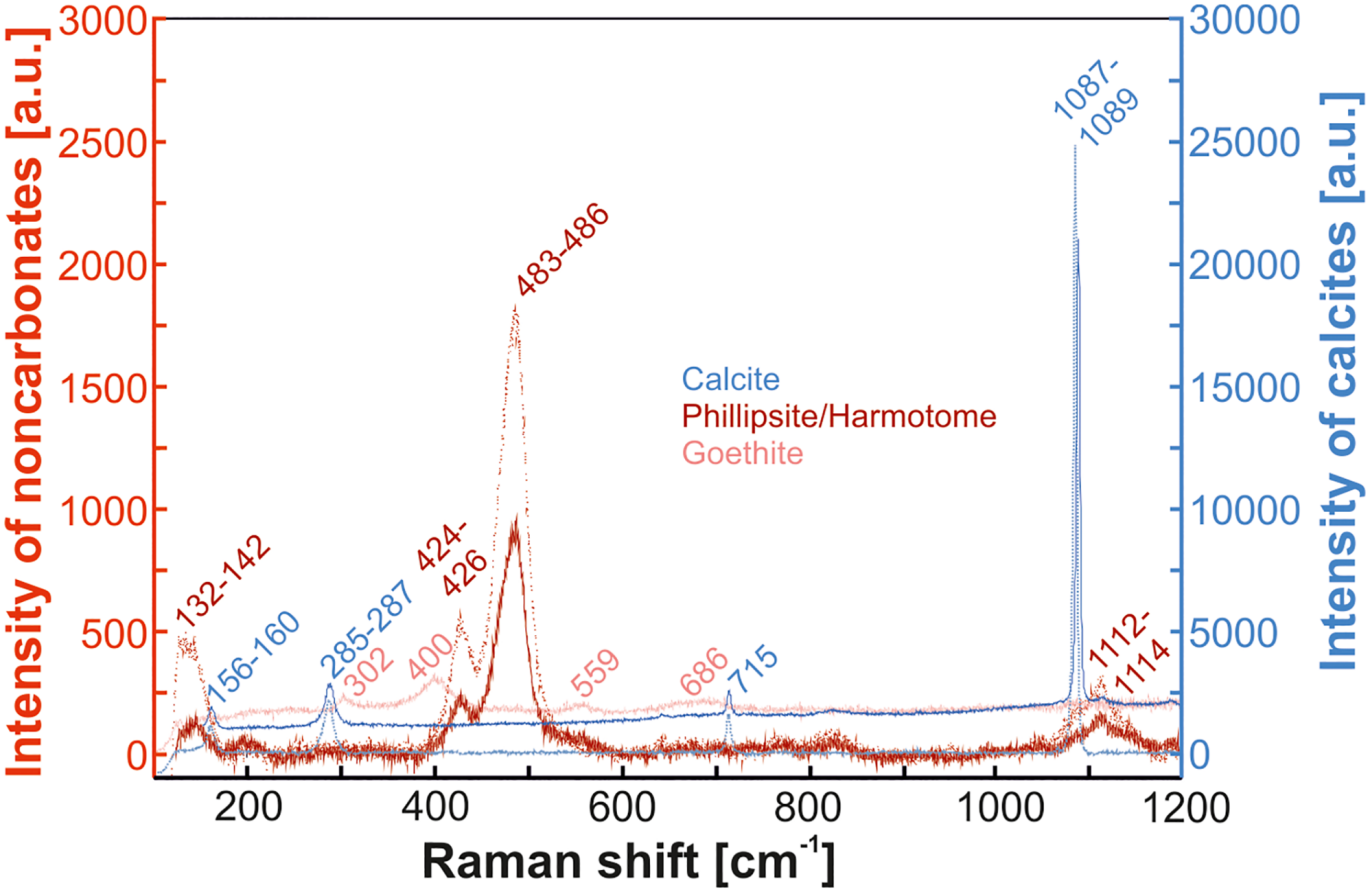
Representative Raman spectra of calcite, phillipsite/harmotome, and goethite. Indicative Raman peak positions are given for each mineral phase. Note the respective intensity axes for carbonates (blue) and noncarbonates (red).

Raman spectra of calcites from the forearc and rear arc display the characteristic minor peak positions at 156–160, 285–287, and 715 cm^−1^, as well as the distinct major peak position at 1,087–1,089 cm^−1^ (Frezzotti et al., 2012) (Figure 3). Zeolites exhibit spectra with major peak positions at 480–490 and 420–430 cm^−1^, typical of phillipsite and/or harmotome (Knight et al., 1989) (Figure 3). Pronounced peak positions at 302 and 400 cm^−1^ and comparatively weak peak positions at 559 and 686 cm^−1^ from opaque minerals characterize goethite (Frezzotti et al., 2012, and references therein) (Figure 3).

### 4.2 Vein Microtextures

Based on microtextures and crystal habits, calcite veins were subdivided into syntaxial, blocky, and antitaxial veins (Bons et al., 2012). Syntaxial veins texturally transition into blocky and antitaxial veins. The textural continuum between syntaxial and antitaxial veins constitutes another vein type termed transitional. A few veins solely consist of microcrystalline calcite (micrite), which also occurs as an additional component in blocky, antitaxial, and transitional calcite veins.

#### 4.2.1 Syntaxial Veins

**Figure 4:**
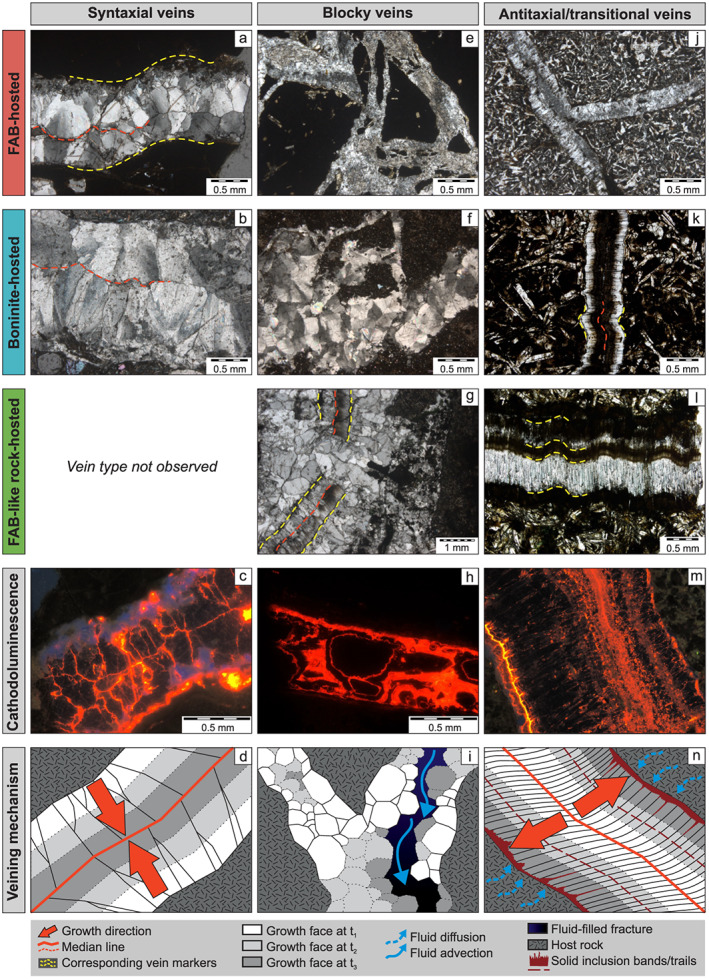
Representative thin section and cathodoluminescence photographs as well as sketches explaining the mode of fracturing, fluid flow, and fracture sealing/mineral growth of syntaxial (a–d), blocky (e–i), and antitaxial/transitional calcite (j–n) veins according to the classification of Bons et al. (2012). The inward growth of syntaxial elongate‐blocky calcites sealed mode I fractures along a median line (d). Blocky calcites grew within fluid‐filled fractures (i). Blocky calcites enclose host rock (e, f) and antitaxial vein fragments (g) and developed Mn‐controlled growth zonations in some cases (h). Preserved median lines and vein margins of antitaxial vein fragments enable the reassembly of corresponding fragments (g). Diffusion‐fed and crystallization pressure‐driven outward growth of antitaxial calcite fibers started at a median line and displaced the host rock (k, n) without fracturing (e.g., Elburg et al., 2002; Meng et al., 2019; Wiltschko & Morse, 2001). Fiber growth faces episodically incorporated solid inclusion bands composed of host rock material. Individual fibers join corresponding markers along the vein margins and indicate the opening trajectory (k, l) (e.g., Durney & Ramsay, 1973; Ramsay & Huber, 1983). The textural continuum between syntaxial and blocky veins is displayed in (c) where the median line is slightly discontinuous (bottom left). In (j) and (m) calcites developed weak growth competition and length‐width ratios ~10 indicative of the transitioning from syntaxial to antitaxial veining (transitional veins).

Syntaxial vein calcites (Figures 4a–4d) developed elongate‐blocky crystal habits, whose length‐width ratios are <10. Calcite growth initiated at both sides of typically few millimeters thick fractures and terminated at a median line. Larger crystals overgrew smaller ones, resulting in an increase in the crystal width and a decrease in the number of grains toward the median line, that is, growth competition (Bons et al., 2012). Median lines and elongate‐blocky crystal habits, however, may be incompletely developed, implying a textural transition into blocky veins. This applies to approximately 50% of all syntaxial veins. CL of syntaxial vein calcites reveals nonluminescent cores and red‐orange luminescent grain boundaries. The occurrence of unambiguous syntaxial veins is restricted to the lower halves of the forearc drill cores (Figure 2). Such well‐developed samples exhibit parallel vein margins, indicating the opening trajectory of the fracture.

#### 4.2.2 Blocky Veins

Blocky calcite veins (Figures 4e–4i) are the dominant vein type and occur independently of depth, host rock, and drill core (Figure 2). They form millimeters to centimeters thick straight veins or irregular networks of branching veins that are composed of randomly distributed equant (blocky) calcite crystals. Blocky calcites enclose rectangular host rock fragments, palagonite shards, and antitaxial vein fragments with preserved median lines, or cement breccias. Argillaceous selvedges that may be accompanied by dark alteration halos mark the vein‐host rock transition. Zeolites either developed early‐stage euhedral crystal habits preceding blocky calcite precipitation or formed anhedral crystals in late‐stage veins crosscutting blocky calcite veins. CL of blocky calcites reveals diversely developed growth zonations. Simple growth zonations are composed of nonluminescent cores and thin red‐orange luminescent grain boundaries. More complex oscillatory growth zonations consist of one to four thin red‐orange luminescent zones in addition to the luminescent grain boundary. Early‐stage and late‐stage zeolites preceding blocky vein calcites and crosscutting them, respectively, display a blue CL.

#### 4.2.3 Antitaxial Veins

Especially in FAB‐like rocks from the Izu‐Bonin rear arc antitaxial veins are prominent structures and approximate the abundance of blocky veins (Figure 2), whereas in the forearc antitaxial veins are rare (boninite) or absent (FAB). Antitaxial veins (Figure 4j–4n) are composed of fibrous calcites with length‐width ratios ≫10. Calcite fibers typically grew from a median line outward, incorporating host rock fragments as solid inclusion bands and trails (Bons et al., 2012). Antitaxial veins either (1) have single median lines in the vein center (symmetrical) or closer to the vein margin (asymmetrical), (2) have multiple median lines often accompanied by micritic infill (multiply (a)symmetrical), or (3) lack median lines (unitaxial). In general, median lines and vein margins are parallel to each other. Antitaxial veins are associated with branching vein systems and occur at different depths. Fragments of antitaxial veins may be incorporated into blocky veins (Figure 4g). Calcite fibers display a high red‐orange CL, which slightly attenuates along fiber long axis.

#### 4.2.4 Transitional Veins

Transitional vein calcites have length‐width ratios ~10, which mark the textural continuum between syntaxial elongate‐blocky (<10) and antitaxial fibrous (>10) (Bons & Jessell, 1997) calcite shapes (Figures 4j and 4m). Similar to antitaxial veins, transitional veins display median lines, which are often spatially associated with micrite, and luminescent inclusion bands. In contrast, slight growth competition of calcite crystals as well as CL characteristics showing nonluminescent cores and red‐orange luminescent grain boundaries conform to the syntaxial vein type. Transitional veins pervade FAB and boninites but lack in FAB‐like rocks from the rear arc where the characteristics of antitaxial veining are well developed (Figure 2).

#### 4.2.5 Micrite Veins

**Figure 5:**
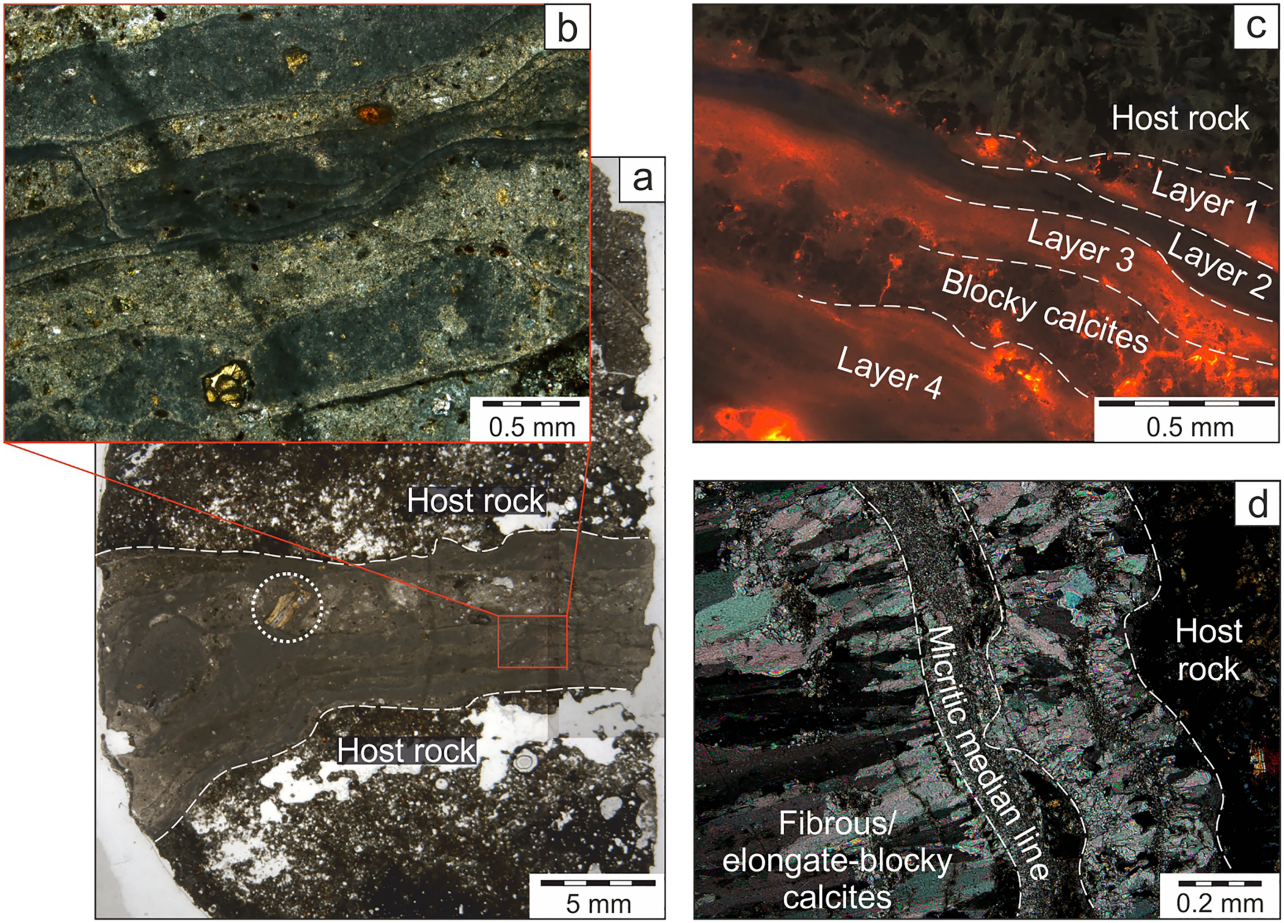
Representative thin section and cathodoluminescence photographs of micrite veins (a–c) and micritic components in transitional veins (d) from the Izu‐Bonin forearc. Compositional differences due to the occurrence of zeolites, palagonite shards (dotted circle), and clay minerals in specific layers cause micritic laminations, which may be distinguished in thin section (a, b) and under cathodoluminescence view (c). Micrite associated with antitaxial and transitional veins concentrates along the median line (d). Sample in (d) is the same sample as shown in Figure 4m.

Veins may be completely (micrite veins) or partly (blocky, antitaxial, and transitional veins) filled with micrite (Figure 5). Micrite veins are often multiply laminated and contain host rock fragments and palagonite shards. Individual layers are distinguishable by their colors comprising shades of white, green, and brown. Nonlaminated micrite occurs in spatial association with blocky veins in which micrite shows a patchy occurrence as well as antitaxial and transitional veins where micrite concentrates along the median lines. They typically display a more whitish color and contain less noncarbonate phases (e.g., zeolites and clay minerals) than do laminated micrites. The occurrence of micrite is restricted to branching vein networks, particularly in the upper levels of the Izu‐Bonin forearc, whereas the rear arc lacks any micritic components (Figure 2). CL of micrite depends on its mineralogical purity. Pure micrite exhibits highly red‐orange luminescent crystals. With increasing amount of noncarbonate phases within micrite accumulations or layers, CL may progressively diminish up to nonluminescence.

### 4.3 Stable Oxygen and Carbon Isotopes

**Figure 6:**
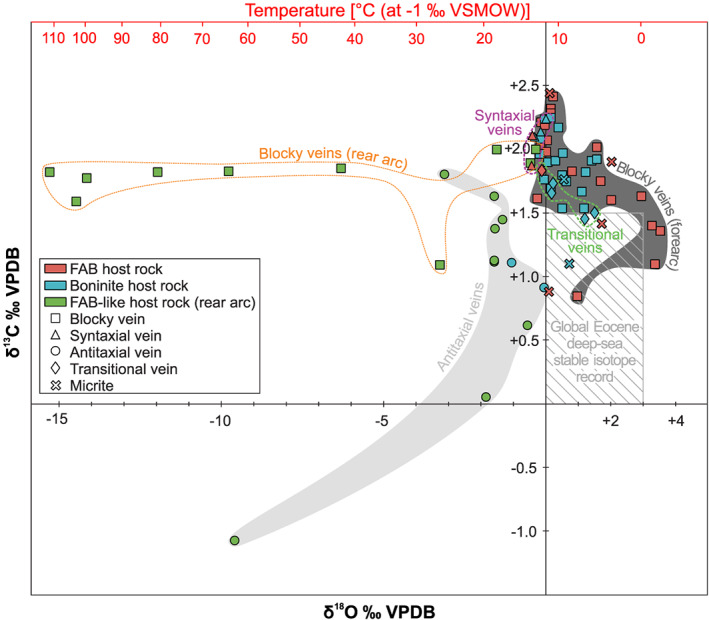
Stable carbon and oxygen isotopic compositions of vein calcites subdivided into drill site (colors) and vein type (symbols). Temperature axis was calculated assuming a seawater composition of −1‰ VSMOW and using the calcite‐water fractionation curve of Friedman and O'Neil (1977). Stable isotope compositions of 14 vein samples were taken from the clumped isotope analyses (see also Table 1). The global Eocene deep‐sea stable isotope record of carbonates refers to Zachos et al. (2001).

Vein calcites have δ^18^O values that range from −15.3‰ to +3.5‰ VPDB and δ^13^C values that vary between −1.1‰ and +2.4‰ VPDB (Figure 6 and Table 1). These oxygen isotopic compositions correspond to δ^18^O formation temperatures (*T*_δ18O_) between 0 and 140 °C using the calcite‐water fractionation curve of Friedman and O'Neil (1977) and assuming equilibrium precipitation from seawater with a range of δ^18^O compositions from −1‰ to +1‰ VSMOW (Table 1). Most samples, however, have δ^18^O values >−0.5‰ VPDB equivalent to formation temperatures <30 °C and δ^13^C values >1.0‰ VPDB, resulting in a point cluster, which overlaps with the field defined by the compilation of Eocene deep‐sea benthic foraminifera (Zachos et al., 2001). Boninite‐hosted vein calcites plot within this point cluster and display a smaller δ^18^O variation (−1.0‰ to +1.6‰ VPDB) than do FAB‐hosted vein calcites (−0.4‰ to +3.5‰ VPDB). Vein calcites from the Izu‐Bonin rear arc define a trend toward significantly negative δ^18^O values as low as −15.3‰ VPDB, which is not found in vein calcites from the other drill sites.

**Table 1:** Carbon and Oxygen Isotopic Compositions of Vein Calcites and Oxygen Isotope Thermometry

Sample	IODP sample ID	Depth (meters below sea level)	Vein type	δ^13^C (‰ VPDB)	δ^18^O (‰ VPDB)	*T*_δ18O_ (°C) (−1‰ VSMOW)	*T*_δ18O_ (°C) (0‰ VSMOW)	*T*_δ18O_ (°C) (+1‰ VSMOW)
BON‐2^a^	352‐U1439C‐3R‐2‐W 107/117	194.23	Micrite	+1.76	+0.57	9.3	13.4	17.7
BON‐3	352‐U1439C‐10R‐1‐W 86/93	261.00	Micrite	+1.10	+0.75	8.5	12.7	16.9
BON‐4	352‐U1439C‐11R‐1‐W 10/14	265.50	Transition	+1.50	+1.51	5.5	9.5	13.7
BON‐4^a^	352‐U1439C‐11R‐1‐W 10/14	265.50	Transition	+1.45	+1.20	6.8	10.8	15.0
BON‐5	352‐U1439C‐12R‐1‐W 93/99	271.33	Blocky	+1.75	+0.66	8.9	13.0	17.3
BON‐6	352‐U1439C‐13R‐1‐W 42/47	281.00	Blocky	+2.17	+0.40	10.0	14.1	18.5
BON‐7^b^	352‐U1439C‐13R‐1‐W 119/128	281.29	Blocky	+1.92	+1.57	5.3	9.3	13.4
BON‐8	352‐U1439C‐19R‐1‐W 68/74	329.48	Blocky	+1.91	+0.29	10.4	14.6	19.0
BON‐9	352‐U1439C‐23R‐1‐W 109/113	359.00	Blocky	+1.91	+1.41	5.9	9.9	14.1
BON‐10	352‐U1439C‐23R‐2‐W 15/21	359.40	Blocky	+1.97	+0.53	9.4	13.6	17.9
BON‐11^b^	352‐U1439C‐25R‐1‐W 18/22	377.78	Blocky	+1.67	+1.12	7.1	11.1	15.3
BON‐12^b^	352‐U1439C‐25R‐2‐W 39/42	379.00	Blocky	+1.54	+1.18	6.8	10.9	15.1
BON‐13^b^	352‐U1439C‐26R‐1‐W 110/120	388.00	Blocky	+1.54	+0.51	9.5	13.7	18.0
BON‐15	352‐U1439C‐29R‐2‐W 71/74	418.48	Blocky	+2.24	−0.02	11.7	15.9	20.4
BON‐16	352‐U1439C‐29R‐4‐W 60/63	421.00	Blocky	+2.25	+0.16	10.9	15.2	19.6
BON‐17^b^	352‐U1439C‐29R‐4‐W 94/96	421.45	Blocky	+1.82	+1.22	6.7	10.7	14.9
BON‐18	352‐U1439C‐30R‐3‐W 36/38	429.00	Blocky	+1.80	+0.52	9.5	13.6	18.0
BON‐19^b^	352‐U1439C‐31R‐1‐W 25/28	436.35	Blocky	+1.76	−0.06	11.9	16.1	20.6
BON‐20	352‐U1439C‐31R‐5‐W 0/5	441.68	Transition	+1.66	+0.18	10.9	15.1	19.5
BON‐20^a^	352‐U1439C‐31R‐5‐W 0/5	441.68	Transition	+1.73	+0.23	10.7	14.9	19.3
BON‐21	352‐U1439C‐32R‐3‐W 113/119	449.52	Blocky	+1.97	−0.20	12.4	16.7	21.2
BON‐22	352‐U1439C‐32R‐4‐W 17/20	450.02	Syntaxial	+2.09	−0.33	13.0	17.3	21.8
BON‐22^a^	352‐U1439C‐32R‐4‐W 17/20	450.02	Syntaxial	+2.14	−0.15	12.2	16.5	21.0
BON‐22^c^	352‐U1439C‐32R‐4‐W 17/20	450.02	Syntaxial	+2.24	+0.02	11.5	15.8	20.2
BON‐23	352‐U1439C‐33R‐2‐W 31/34	457.00	Blocky	+2.09	−0.18	12.4	16.6	21.1
BON‐24^b^	352‐U1439C‐35R‐2‐W 6/13	466.81	Blocky	+1.90	+0.07	11.3	15.6	20.0
BON‐25^b^	352‐U1439C‐35R‐2‐W 81/90	467.00	Blocky	+1.69	+0.16	10.9	15.2	19.6
BON‐26	352‐U1439C‐41R‐1‐W 90/93	515.00	Antitaxial	+0.91	−0.06	11.9	16.1	20.6
BON‐26^a^	352‐U1439C‐41R‐1‐W 90/93	515.00	Antitaxial	+1.11	−1.04	16.0	20.5	25.1
BON‐26^c^	352‐U1439C‐41R‐1‐W 90/93	515.00	Antitaxial	+0.91	−0.06	11.9	16.1	20.6
FAB‐1^b^	352‐U1440B‐12R‐1‐W 145/149	165.09	Micrite	+0.88	+0.11	11.2	15.4	19.8
FAB‐2^b^	352‐U1440B‐12R‐1‐W 145/149	165.09	Blocky	+1.10	+3.38	−1.4	2.2	6.1
FAB‐3^b^	352‐U1440B‐12R‐2‐W 61/63	165.79	Blocky	+1.36	+3.54	−2.0	1.6	5.4
FAB‐4^b^	352‐U1440B‐17R‐1‐W 58/63	212.83	Blocky	+1.75	+1.69	4.8	8.8	12.9
FAB‐4^c^	352‐U1440B‐17R‐1‐W 58/63	212.83	Blocky	+1.60	+2.03	3.5	7.4	11.5
FAB‐5^b^	352‐U1440B‐18R‐1‐W 19/30	220.51	Blocky	+0.84	+0.98	7.6	11.7	15.9
FAB‐6^b^	352‐U1440B‐18R‐1‐W 91/102	221.23	Micrite	+1.90	+2.04	3.5	7.4	11.5
FAB‐7	352‐U1440B‐21R‐1‐W 61/67	237.02	Syntaxial	+2.11	−0.40	13.3	17.6	22.1
FAB‐8	352‐U1440B‐22R‐1‐W 10/14	241.54	Micrite	+2.44	+0.13	11.1	15.3	19.7
FAB‐9	352‐U1440B‐23R‐1‐W 0/5	246.16	Blocky	+2.28	+0.17	10.9	15.1	19.5
FAB‐9^a^	352‐U1440B‐23R‐1‐W 0/5	246.16	Blocky	+2.32	+0.15	11.0	15.2	19.6
FAB‐11	352‐U1440B‐23R‐1‐W 40/44	246.54	Blocky	+2.42	+0.22	10.7	14.9	19.3
FAB‐12	352‐U1440B‐24R‐1‐W 38/42	251.51	Blocky	+2.21	−0.16	12.3	16.6	21.0
FAB‐13	352‐U1440B‐28R‐1‐W 16/21	290.23	Transition	+1.84	−0.12	12.1	16.4	20.8
FAB‐14	352‐U1440B‐31R‐1‐W 64/70	319.88	Syntaxial	+1.87	−0.43	13.4	17.7	22.3
FAB‐15	352‐U1440B‐32R‐1‐W 26/34	329.34	Blocky	+1.98	+0.02	11.5	15.8	20.2
FAB‐16	352‐U1440B‐35R‐1‐W 32/37	358.56	Blocky	+1.61	−0.25	12.7	16.9	21.4
FAB‐17	352‐U1441A‐14R‐1‐W 103/108	122.91	Blocky	+1.63	+2.96	0.1	3.8	7.7
FAB‐18^a^	352‐U1441A‐14R‐1‐W 129/131	123.09	Blocky	+1.40	+3.29	−1.1	2.6	6.4
FAB‐19	352‐U1441A‐15R‐1‐W 58/66	132.16	Blocky	+2.02	+1.58	5.3	9.2	13.4
FAB‐20^b^	352‐U1441A‐15R‐1‐W 58/66	132.16	Micrite	+1.41	+1.74	4.7	8.6	12.7
FAB‐21	352‐U1441A‐17R‐1‐W 21/23	151.25	Blocky	+2.19	−0.03	11.7	16.0	20.4
FAB‐22	352‐U1441A‐18R‐1‐W 17/21	160.90	Blocky	+2.07	+0.04	11.4	15.7	20.1
FAB‐22^a^	352‐U1441A‐18R‐1‐W 17/21	160.90	Blocky	+1.83	+0.81	8.3	12.4	16.7
FAB‐23	352‐U1441A‐21R‐1‐W 34/37	190.27	Blocky	+2.02	−0.17	12.3	16.6	21.1
ASB‐1^a^	351‐U1438E‐71R‐2‐W 29/34	1,471.83	Blocky	+1.78	−14.17	99.6	109.2	119.7
ASB‐1^b^	351‐U1438E‐71R‐2‐W 29/34	1,471.83	Blocky	+1.82	−11.98	80.5	88.7	97.5
ASB‐2^b^	351‐U1438E‐71R‐2‐W 48/53	1,472.00	Blocky	+2.00	−0.30	12.9	17.2	21.7
ASB‐3^a^	351‐U1438E‐71R‐3‐W 67/74	1,473.40	Blocky	+1.83	−9.77	63.9	71.0	78.6
ASB‐3^b^	351‐U1438E‐71R‐3‐W 67/74	1,473.40	Blocky	+1.85	−6.31	42.2	48.0	54.1
ASB‐4^a^	351‐U1438E‐72R‐1‐W 6/12	1,474.76	Antitaxial	+1.80	−3.12	25.5	30.5	35.6
ASB‐4^b^	351‐U1438E‐72R‐1‐W 6/12	1,474.76	Antitaxial	+1.63	−1.59	18.5	23.0	27.8
ASB‐5^a^	351‐U1438E‐72R‐1‐W 6/12	1,474.76	Blocky	+1.82	−15.30	110.7	121.3	132.8
ASB‐5^b^	351‐U1438E‐72R‐1‐W 6/12	1,474.76	Blocky	+1.59	−14.48	102.5	112.4	123.2
ASB‐6	351‐U1438E‐72R‐1‐W 109/112	1,475.80	Blocky	+2.00	−1.51	18.1	22.7	27.4
ASB‐7	351‐U1438E‐72R‐1‐W 109/112	1,475.80	Blocky	+1.12	−1.59	18.5	23.0	27.8
ASB‐8^b^	351‐U1438E‐73R‐1‐W 111/115	1,485.51	Antitaxial	+0.62	−0.57	14.0	18.4	22.9
ASB‐9^a^	351‐U1438E‐73R‐2‐W 60/64	1,486.19	Antitaxial	+1.45	−1.33	17.3	21.8	26.5
ASB‐9^b^	351‐U1438E‐73R‐2‐W 60/64	1,486.19	Antitaxial	+1.37	−1.57	18.4	22.9	27.7
ASB‐10	351‐U1438E‐73R‐2‐W 60/64	1,486.19	Blocky	+1.09	−3.26	26.2	31.2	36.4
ASB‐11	351‐U1438E‐73R‐2‐W 60/64	1,486.19	Antitaxial	+1.13	−1.60	18.5	23.1	27.9
ASB‐12^a^	351‐U1438E‐78R‐4‐W 12/16	1,519.73	Antitaxial	−1.07	−9.60	62.8	69.8	77.3
ASB‐13	351‐U1438E‐79R‐1‐W 82/86	1,524.50	Antitaxial	+0.05	−1.84	19.6	24.2	29.0
ASB‐15	351‐U1438E‐82R‐2‐W 43/52	1,553.77	Blocky	+1.90	−0.48	13.6	18.0	22.5

*Note*. The oxygen isotope temperature *T*_δ18O_ was calculated after the calcite‐water fractionation curve of Friedman and O'Neil (1977) assuming equilibrium calcite precipitation from seawater with a range of δ^18^O compositions from −1 to +1‰ VSMOW. Analytical precision of stable isotope measurements was better than ±0.15‰ VPDB for δ^18^O and ±0.1‰ VPDB for δ^13^C. ASB = FAB‐like rock‐hosted veins from the Amami‐Sankaku basin (Site U1438); BON = boninite‐hosted veins (Site U1439); FAB = forearc basalt‐hosted veins (FAB‐1 to FAB‐16 from Site U1440 and FAB‐17 to FAB‐23 from Site U1441).

a Stable isotope values taken from clumped isotope analysis. See also Table 2 for details.

b Samples analyzed at the JR‐AquaConSol GmbH, Graz (Austria). All other samples were analyzed at the NAWI Graz Geocenter, Institute of Earth Sciences, University of Graz (Austria).

c Repeated analysis of separately drilled powders from the same sample.

**Figure 7:**
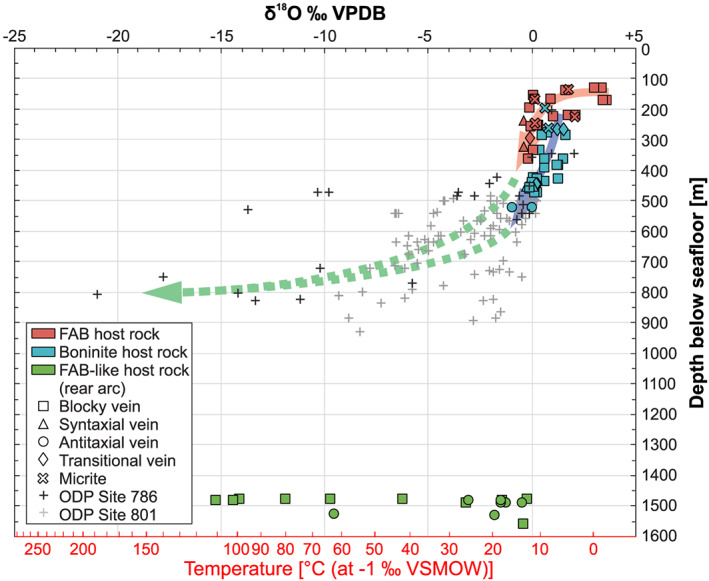
Correlation between depth below seafloor and δ^18^O composition of vein calcites from the forearc and rear arc. Stable isotope compositions of 14 vein samples were taken from the clumped isotope analyses (see also Table 1). The δ^18^O compositions of this study are compared with δ^18^O compositions of vein calcites from ODP Site 786 in the northern Izu‐Bonin forearc (Alt et al., 1998) and ODP Site 801 in the Western Pacific Pigafetta basin (Alt & Teagle, 2003). Temperature axis was calculated as in Figure 6.

A general trend toward more negative δ^18^O values with depth below seafloor is observed in vein calcites from the forearc (Figure 7). Oxygen isotopes of vein calcites from Ocean Drilling Program (ODP) Site 786 in the northern Izu‐Bonin forearc (Alt et al., 1998) and ODP Site 801 in the Western Pacific Pigafetta basin (Alt & Teagle, 2003) extend this trend to greater depths. Below 450−550 m below seafloor, oxygen isotopic variation increases significantly including also vein calcites from the rear arc.

Independently of the host rock, δ^18^O and δ^13^C compositions define fields related to the vein type (Figure 6). The lowest δ^18^O compositions (<−3.2‰ VPDB) predominantly refer to blocky vein calcites. Syntaxial vein calcites plot uniformly within the point cluster defined by samples with δ^13^C >1.0‰ VPDB and δ^18^O >−0.5‰ VPDB. They have rather high δ^13^C values ≥1.9‰ VPDB and well‐constrained δ^18^O compositions between 0.0‰ and −0.4‰ VPDB, resulting in a narrow field for this vein type. The range of δ^13^C compositions of antitaxial vein calcites includes the lowest δ^13^C values (<+1.8‰ VPDB) of all samples and plots below the field of syntaxial veins. Transitional veins marking the textural continuum between syntaxial and antitaxial veins span a field whose δ^13^C values overlap with the upper range of antitaxial veins and the lower range of syntaxial veins. Stable isotope compositions of separately drilled powders of the same sample show a good consistency in the carbon isotope composition, but some samples, especially from the rear arc, reveal strong inhomogeneities in δ^18^O (Table 1).

### 4.4 Clumped Isotopes

**Figure 8:**
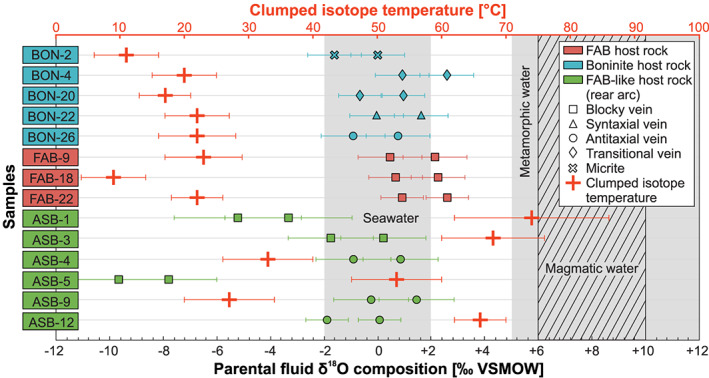
Clumped isotope temperatures and parental fluid δ^18^O compositions of vein calcites subdivided into drill site (colors) and vein type (symbols). Clumped isotope temperatures (red crosses) are calculated using the inorganic calcite calibration of Kele et al. (2015). By using this temperature and the calibrations of Kim and O'Neil (1997) as well as Coplen (2007), the parental fluid δ^18^O compositions (colored vein type symbols) were calculated. Temperature error bars refer to the 95% confidence interval. Isotopic compositions of reference fields are taken from Hoefs (2015).

The analyzed vein calcites reveal consistently low clumped isotope temperatures (*T*_Δ47_) ranging from 9 ± 5 to 74 ± 12 °C (Figures 8 and Table 2). The mean *T*_Δ47_ of 33 °C separates these temperatures into two groups. The higher *T*_Δ47_ (>33 °C) belong to veins from the Izu‐Bonin rear arc, whereas the lower *T*_Δ47_ (<33 °C) refer to veins hosted in FAB and boninites from the Izu‐Bonin forearc.

**Table 2:** Clumped Isotope Temperatures and Parental Fluid δ^18^O Values of Vein Calcites

Sample	IODP sample ID	Depth (meters below sea level)	Vein type	δ^13^C (‰ VPDB)	δ^18^O (‰ VPDB)	Δ_47_ (‰)	Number of replicates	*T*_Δ47_ (°C)	Parental fluid δ^18^O (‰ VSMOW)^a^	Parental fluid δ^18^O (‰ VSMOW)^b^
BON‐2	352‐U1439C‐3R‐2‐W 107/117	194.23	Micrite	+1.76 ± 0.02	+0.57 ± 0.06	0.726 ± 0.030	10	11 ± 5	−0.01 ± 1.0	−1.61 ± 1.0
BON‐4	352‐U1439C‐11R‐1‐W 10/14	265.50	Transitional	+1.45 ± 0.01	+1.20 ± 0.05	0.689 ± 0.015	6	20 ± 5	+2.57 ± 1.0	+0.90 ± 1.0
BON‐20	352‐U1439C‐31R‐5‐W 0/5	441.68	Transitional	+1.73 ± 0.01	+0.23 ± 0.03	0.702 ± 0.019	10	17 ± 4	+0.96 ± 0.8	−0.69 ± 0.8
BON‐22	352‐U1439C‐32R‐4‐W 17/20	450.02	Syntaxial	+2.14 ± 0.04	−0.15 ± 0.12	0.683 ± 0.026	11	22 ± 5	+1.64 ± 1.0	−0.05 ± 1.0
BON‐26	352‐U1439C‐41R‐1‐W 90/93	515.00	Antitaxial	+1.11 ± 0.02	−1.04 ± 0.04	0.684 ± 0.031	10	22 ± 6	+0.74 ± 1.2	−0.94 ± 1.2
FAB‐9	352‐U1440B‐23R‐1‐W 0/5	246.16	Blocky	+2.32 ± 0.01	+0.15 ± 0.04	0.681 ± 0.024	7	23 ± 6	+2.14 ± 1.2	+0.45 ± 1.2
FAB‐18	352‐U1441A‐14R‐1‐W 129/131	123.09	Blocky	+1.40 ± 0.01	+3.29 ± 0.02	0.730 ± 0.022	8	9 ± 5	+2.26 ± 1.0	+0.67 ± 1.0
FAB‐22	352‐U1441A‐18R‐1‐W 17/21	160.90	Blocky	+1.83 ± 0.02	+0.81 ± 0.02	0.684 ± 0.015	7	22 ± 4	+2.60 ± 0.8	+0.91 ± 0.8
ASB‐1	351‐U1438E‐71R‐2‐W 29/34	1,471.83	Blocky	+1.78 ± 0.06	−14.17 ± 0.53	0.540 ± 0.029	7	74 ± 12	−3.33 ± 2.4	−5.33 ± 2.4
ASB‐3	351‐U1438E‐71R‐3‐W 67/74	1,473.40	Blocky	+1.83 ± 0.01	−9.77 ± 0.02	0.554 ± 0.019	7	68 ± 8	+0.20 ± 1.6	−1.77 ± 1.6
ASB‐4	351‐U1438E‐72R‐1‐W 6/12	1,474.76	Antitaxial	+1.80 ± 0.02	−3.12 ± 0.12	0.647 ± 0.019	5	33 ± 7	+0.85 ± 1.4	−0.91 ± 1.4
ASB‐5	351‐U1438E‐72R‐1‐W 6/12	1,474.76	Blocky	+1.82 ± 0.01	−15.30 ± 0.09	0.591 ± 0.032	10	53 ± 9	−7.80 ± 1.8	−9.67 ± 1.8
ASB‐9	351‐U1438E‐73R‐2‐W 60/64	1,486.19	Antitaxial	+1.45 ± 0.01	−1.33 ± 0.04	0.666 ± 0.029	8	27 ± 7	+1.47 ± 1.4	−0.25 ± 1.4
ASB‐12	351‐U1438E‐78R‐4‐W 12/16	1,519.70	Antitaxial	−1.07 ± 0.02	−9.60 ± 0.10	0.558 ± 0.016	12	66 ± 4	+0.06 ± 0.8	−1.90 ± 0.8

*Note*. Reported errors refer to standard deviation (δ^13^C, δ^18^O, and Δ_47_) and 95% confidence interval (*T*_Δ47_). ASB = FAB‐like rock‐hosted veins from the Amami‐Sankaku basin (Site U1438); BON = boninite‐hosted veins (Site U1439); FAB = forearc basalt‐hosted veins (FAB‐9 from Site U1440 and FAB‐18 and FAB‐22 from Site U1441).

a Parental fluid δ^18^O value using calibrations of Kele et al. (2015) and Kim and O'Neil (1997).

b Parental fluid δ^18^O value using calibrations of Kele et al. (2015) and Coplen (2007).

Independently of vein type and host rock, most parental fluid δ^18^O compositions (Figure 8 and Table 2) fall within the conservatively estimated seawater range (−2‰ to +2‰ VSMOW) (Hoefs, 2015) or are slightly enriched relative to seawater (up to +2.6‰ VSMOW). Two pairs of values of blocky veins from the Izu‐Bonin rear arc, however, show significantly lower calculated compositions than seawater (−3.3‰ to −9.7‰ VSMOW), whereas another blocky vein from the same location falls within the seawater field (−1.8‰ to +0.2‰ VSMOW). Stable and clumped isotopic analyses of separately drilled powders of the same sample yielded similar δ^18^O and δ^13^C values for most vein calcites, but deviations from each other by up to 3.5‰ VPDB especially exist for vein calcites from the rear arc (Tables 1 and 2).

### 4.5 Radiogenic Strontium Isotopes

**Figure 9:**
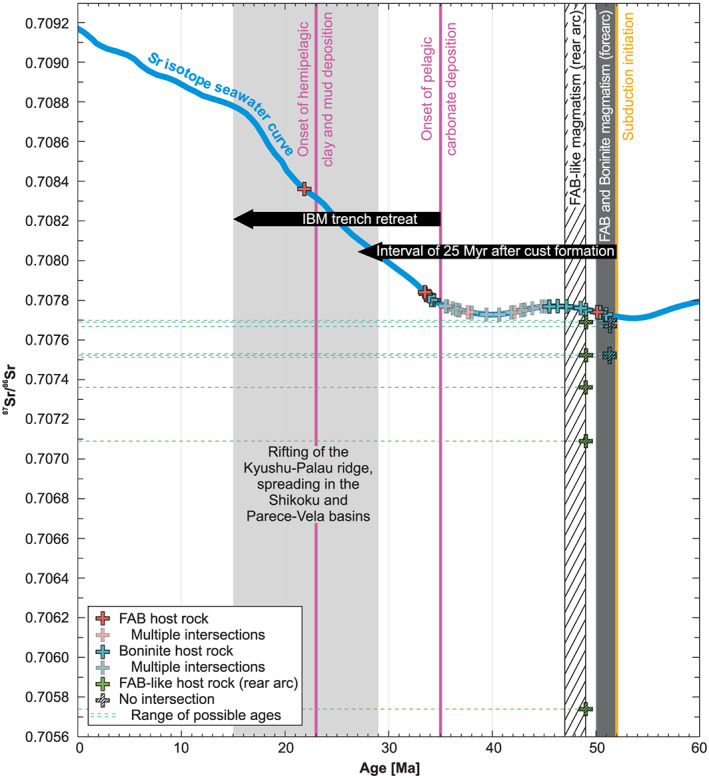
^87^Sr/^86^Sr ratios of vein calcites subdivided into host rock (colors) and the Sr isotope seawater curve (McArthur et al., 2001). Some samples intersect the curve multiple times. Except for the oldest intersection, these multiple intersections of individual samples are indicated by pale symbols. Dashed samples represent vein calcites lacking any intersection with the seawater curve. Their maximum ages, which are constrained by the time of host rock emplacement, are plotted. Horizontal dashed lines indicate that these vein calcites may have formed at any time between host rock emplacement and today. Geological background information refers to Arculus, Ishizuka, Bogus, Gurnis, et al. (2015), Arculus, Ishizuka, Bogus, & the Expedition 351 Scientists (2015), Brandl et al. (2017), Faccenna et al. (2018), Hickey‐Vargas et al. (2018), Ishizuka, Tani, et al. (2011), Ishizuka et al. (2018), Okino et al. (1998), Sdrolias et al. (2004), Reagan et al. (2013), Reagan et al. (2019), and Robertson et al. (2018). The interval of 25 Myr after crust formation refers to the general time interval during which >80% of secondary mineralization of oceanic crust is complete (e.g., Coogan & Gillis, 2018, and references therein).

^87^Sr/^86^Sr ratios vary from 0.70574 to 0.70836, but most samples cluster between 0.70770 and 0.70784 (Table 3). This cluster intersects the Sr isotope seawater curve (McArthur et al., 2001) for the Eocene to present day between ~52 and ~33 Ma (Figure 9). The sigmoidal shape and shallow slope of the Sr isotope seawater curve until ~35 Ma leads to multiple intersections. One higher ^87^Sr/^86^Sr ratio (0.70836) refers to a single FAB‐hosted blocky vein calcite that intersects the seawater curve at ~22 Ma. Lower ^87^Sr/^86^Sr ratios (<0.70770) lack any intersection with the seawater curve and refer to all veins from the Izu‐Bonin rear arc as well as a few blocky and antitaxial veins hosted in boninites from the Izu‐Bonin forearc. Antitaxial and transitional vein calcites mostly have ^87^Sr/^86^Sr ratios below the seawater curve, whereas blocky vein calcites cover the whole ^87^Sr/^86^Sr range of the sample suite.

**Table 3:** Strontium Isotopes of Vein Calcites

Sample	IODP sample ID	Depth (meters below sea level)	Vein type	^87^Sr/^86^Sr
BON‐1	352‐U1439C‐3R‐1‐W 33/37	192.13	Micrite	0.70775 ± 0.00001
BON‐2	352‐U1439C‐3R‐2‐W 107/117	194.23	Micrite	0.70782 ± 0.00001
BON‐4	352‐U1439C‐11R‐1‐W 10/14	265.50	Transition	0.70777 ± 0.00001
BON‐5	352‐U1439C‐12R‐1‐W 93/99	271.33	Blocky	0.70774 ± 0.00001
BON‐7	352‐U1439C‐13R‐1‐W 119/128	281.29	Blocky	0.70780 ± 0.00001
BON‐8	352‐U1439C‐19R‐1‐W 68/74	329.48	Blocky	0.70780 ± 0.00001
BON‐10	352‐U1439C‐23R‐2‐W 15/21	359.40	Blocky	0.70770 ± 0.00001
BON‐11	352‐U1439C‐25R‐1‐W 18/22	377.78	Blocky	0.70751 ± 0.00001
BON‐14	352‐U1439C‐26R‐2‐W 9/11	388.89	Blocky	0.70774 ± 0.00001
BON‐14^a^	352‐U1439C‐26R‐2‐W 9/11	388.89	Blocky	0.70773 ± 0.00001
BON‐15	352‐U1439C‐29R‐2‐W 71/74	418.48	Blocky	0.70776 ± 0.00001
BON‐17	352‐U1439C‐29R‐4‐W 94/96	421.45	Blocky	0.70777 ± 0.00001
BON‐19	352‐U1439C‐31R‐1‐W 25/28	436.35	Blocky	0.70767 ± 0.00001
BON‐20	352‐U1439C‐31R‐5‐W 0/5	441.68	Transition	0.70753 ± 0.00001
BON‐21	352‐U1439C‐32R‐3‐W 113/119	449.52	Blocky	0.70775 ± 0.00001
BON‐24	352‐U1439C‐35R‐2‐W 6/13	466.81	Blocky	0.70777 ± 0.00001
FAB‐4	352‐U1440B‐17R‐1‐W 58/63	212.83	Blocky	0.70783 ± 0.00001
FAB‐7	352‐U1440B‐21R‐1‐W 61/67	237.02	Syntaxial	0.70774 ± 0.00001
FAB‐10	352‐U1440B‐23R‐1‐W 6/9	246.19	Blocky	0.70780 ± 0.00001
FAB‐18	352‐U1441A‐14R‐1‐W 129/131	123.09	Blocky	0.70836 ± 0.00001
FAB‐22	352‐U1441A‐18R‐1‐W 17/21	160.90	Blocky	0.70784 ± 0.00001
ASB‐1	351‐U1438E‐71R‐2‐W 29/34	1,471.83	Blocky	0.70736 ± 0.00001
ASB‐4	351‐U1438E‐72R‐1‐W 6/12	1,474.76	Antitaxial	0.70767 ± 0.00001
ASB‐4^b^	351‐U1438E‐72R‐1‐W 6/12	1,474.76	Antitaxial	0.70767 ± 0.00001
ASB‐5	351‐U1438E‐72R‐1‐W 6/12	1,474.76	Blocky	0.70574 ± 0.00001
ASB‐8	351‐U1438E‐73R‐1‐W 111/115	1,485.51	Antitaxial	0.70709 ± 0.00001
ASB‐9	351‐U1438E‐73R‐2‐W 60/64	1,486.19	Antitaxial	0.70771 ± 0.00001
ASB‐9^b^	351‐U1438E‐73R‐2‐W 60/64	1,486.19	Antitaxial	0.70771 ± 0.00001
ASB‐14	351‐U1438E‐82‐R1‐W 37/43	1,552.37	Blocky	0.70752 ± 0.00002

*Note*. ASB = FAB‐like rock‐hosted veins from the Amami‐Sankaku basin (Site U1438); BON = boninite‐hosted veins (Site U1439); FAB = forearc basalt‐hosted veins (FAB‐4 to FAB‐10 from Site U1440 and FAB‐18 and FAB‐22 from Site U1441).

a Repeated analysis of a separately drilled powder from the same sample.

b Repeated analysis on the same sample solution.

**Figure 10:**
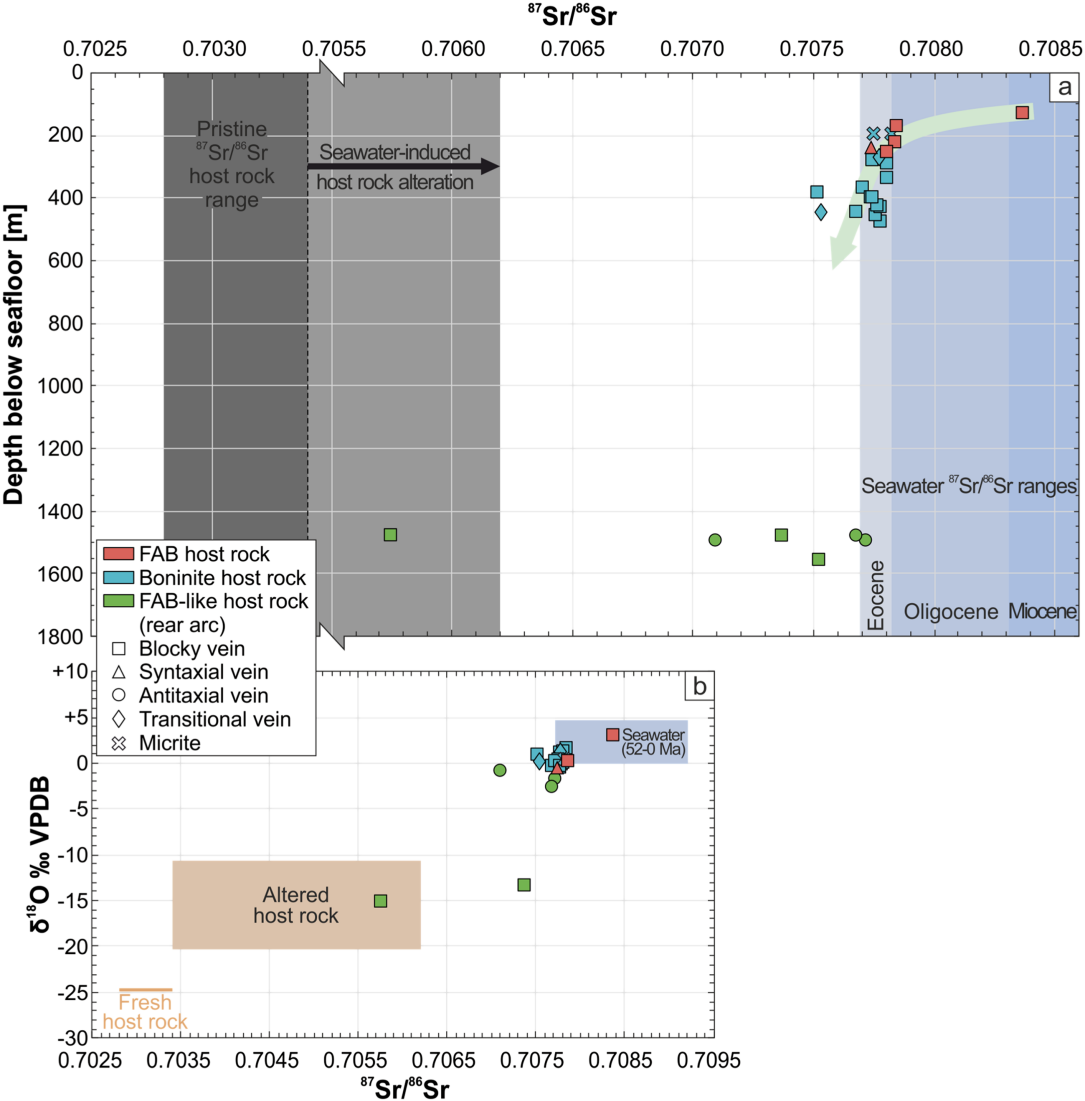
(a) Negative correlation (green arrow) between ^87^Sr/^86^Sr ratios and depth of vein calcites. Shallow FAB‐hosted vein calcites have the highest ^87^Sr/^86^Sr ratios, which transition into lower values of deeper boninite‐hosted vein calcites. The lowest ^87^Sr/^86^Sr ratios are recorded by deep vein calcites from the rear arc. Seawater reference values for the Eocene, Oligocene, and Miocene refer to McArthur et al. (2001). The range of host rock reference values comprises boninites, FAB, and FAB‐like rocks, which were recovered during IODP Expedition 351, submersible dives, and seafloor dredging (Ishizuka et al., 2006; Ishizuka, Taylor, et al., 2011; Reagan et al., 2010; Yogodzinski et al., 2018). Yogodzinski et al. (2018) define ^87^Sr/^86^Sr ratios <0.70340 as representative for preserved magmatic compositions. Higher ^87^Sr/^86^Sr ratios are probably the result of host rock‐seawater interaction (Yogodzinski et al., 2018). Boninites tend to have higher ^87^Sr/^86^Sr ratios than FAB (Reagan et al., 2010). Note that the *x*‐axis is compressed in order to show the full range of ^87^Sr/^86^Sr ratios. (b) Positive correlation between ^87^Sr/^86^Sr ratios and δ^18^O isotopic compositions of vein calcites. The sample suite defines a trend from a seawater end‐member to an altered host rock end‐member. Host rock reference values refer to Eiler et al. (2000), Reagan et al. (2010), and Staudigel et al. (1995). Seawater reference values refer to McArthur et al. (2001) and Zachos et al. (2001).

In Figures 10a and 10b ^87^Sr/^86^Sr ratios are plotted against depth below seafloor and δ^18^O isotopic compositions, respectively. ^87^Sr/^86^Sr ratios of forearc vein calcites decrease with increasing depth (Figure 10a). This trend is defined by shallow FAB‐hosted vein calcites with high ^87^Sr/^86^Sr ratios and deeper boninite‐hosted vein calcites with lower ^87^Sr/^86^Sr ratios. ^87^Sr/^86^Sr ratios and δ^18^O isotopic compositions of the sample suite are positively correlated (Figure 10b). The sample suite defines a trend from Eocene seawater toward altered host rock compositions. Vein calcites from the Izu‐Bonin rear arc show the most host rock‐like compositions.

### 4.6 Rare Earth Elements and Yttrium

**Figure 11:**
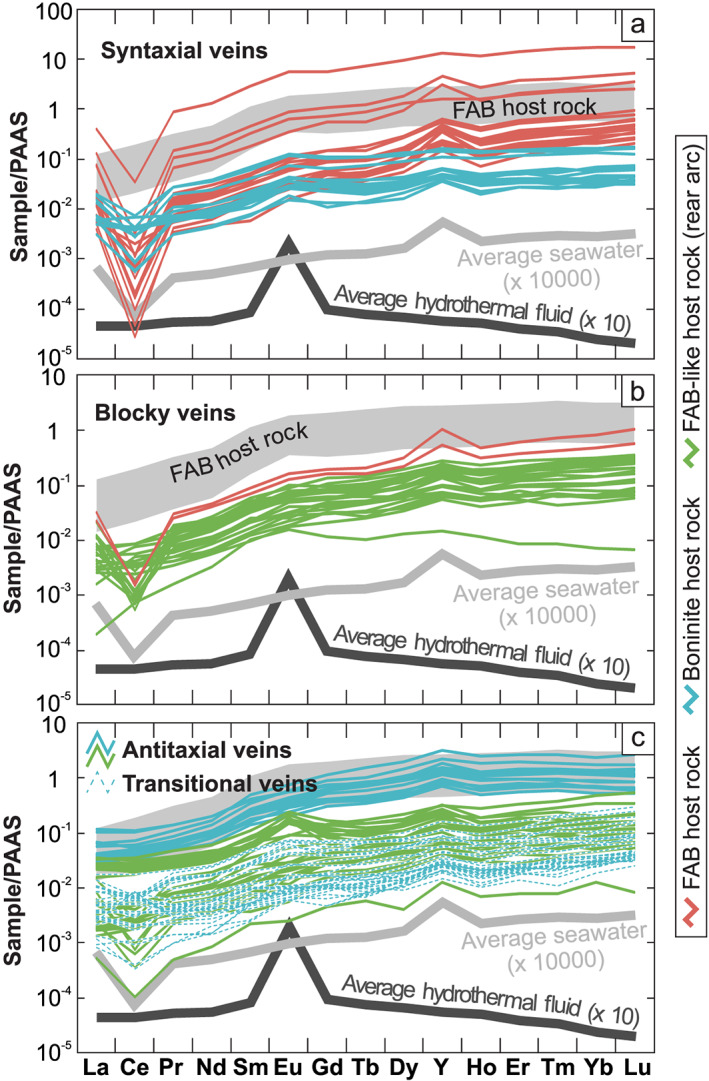
Post‐Archean Australian Shale (PAAS)‐normalized (McLennan, 1989) REE+Y distribution patterns subdivided into drill site (colors) and (a) syntaxial, (b) blocky, and (c) antitaxial/transitional veins. Host rock ranges, average hydrothermal fluid, and average seawater reference values refer to Shervais et al. (2019), Bau and Dulski (1999), and Zhang and Nozaki (1996), respectively. REE+Y data on boninites from the Izu‐Bonin trench are limited. They show similar PAAS‐normalized patterns as FAB but have lower heavy rare earth element concentrations (e.g., Reagan et al., 2010).

PAAS‐normalized (McLennan, 1989) REE+Y concentrations of vein calcites exhibit heavy REE‐enriched distribution patterns with negative to slightly positive Ce, positive Eu, and positive Y anomalies (Figure 11). Vein calcites have higher concentrations in REE+Y than do seawater (Zhang & Nozaki, 1996) and hydrothermal fluids (Bau & Dulski, 1999). Here the term hydrothermal fluid is defined as seawater that entered the oceanic crust and interacted at depth with rocks under temperatures up to >400 °C. This involves the enrichment of dissolved metals and gases in the fluid phase (Koschinsky, 2014). Some samples have REE+Y concentrations as high as those of FAB (Shervais et al., 2019).

Calculated PAAS‐normalized Ce anomalies (Ce/Ce*_PAAS_) range from 0.0 to 3.3. Especially, antitaxial and transitional vein calcites show a high proportion of positive (>1) Ce/Ce*_PAAS_ up to 3.3, whereas blocky and syntaxial vein calcites are dominated by negative (<1) Ce/Ce*_PAAS_ (0.6 and 0.3 on average, respectively). Calculated PAAS‐normalized Eu anomalies (Eu/Eu*_PAAS_) span from seawater‐like values (1.0 ± 0.5) to significantly increased values up to 2.9. This range is based on antitaxial vein calcites from the rear arc and involves the lowest (<0.8) as well as highest Eu/Eu*_PAAS_ (>1.7). Blocky and syntaxial vein calcites have Eu/Eu*_PAAS_ ≤2.0. Most Y/Ho ratios are <50 and cluster around the minimum value of seawater (36) (Tostevin et al., 2016). This cluster comprises most blocky, antitaxial, and transitional vein calcites as well as some syntaxial vein calcites. Other syntaxial vein calcites, particularly those hosted in FAB, display the highest Y/Ho ratios (50–75).

**Figure 12:**
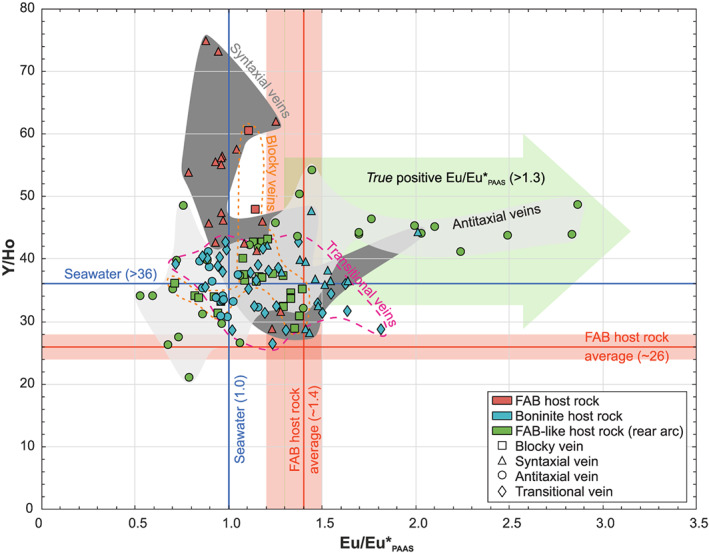
Y/Ho and PAAS‐normalized Eu anomalies (Eu/Eu*_PAAS_) subdivided into drill site (colors) and vein type (symbols). Due to the inheritance of Eu anomalies from shale normalization relative to chondrite normalization, only Eu/Eu*_PAAS_ >1.3 are considered as true Eu anomalies (green arrow). Host rock and seawater reference values refer to Shervais et al. (2019) and Zhang and Nozaki (1996), respectively. REE+Y data on boninites from the Izu‐Bonin trench are limited and incomplete. Available Y/Ho ratios and Eu/Eu*_PAAS_ tend to be slightly higher in boninites (e.g., Reagan et al., 2010). Hydrothermal fluids typically have Y/Ho ratios from 28 to 45 and Eu/Eu*_PAAS_ >7 (Bau & Dulski, 1999).

Eu/Eu*_PAAS_ and Y/Ho ratios of each sample are plotted in Figure 12. The subdivision into vein type and host rock reveals specific compositional fields for the different vein types as well as distinct fields for veins with the same host rock. The fields for antitaxial veins and veins from the Izu‐Bonin rear arc are indistinguishable. Both are characterized by a significant variation of Eu/Eu*_PAAS_ and a small range of Y/Ho ratios. In contrast, the fields for veins from the forearc and fields for syntaxial and blocky veins show a higher variation in Y/Ho, whereas the range of Eu/Eu*_PAAS_ is restricted. Transitional veins marking the textural continuum between antitaxial and syntaxial veins display the low Y/Ho ratios of antitaxial veins and the restricted Eu/Eu*_PAAS_ of syntaxial veins. All fields overlap in the center of the plot (Y/Ho = 36 ± 8, Eu/Eu*_PAAS_ = 1 ± 0.5) where the point density is highest.

## 5 Discussion

### 5.1 Mode of Fracturing, Fluid Flow, and Fracture Sealing/Mineral Growth

#### 5.1.1 Syntaxial Veins

Most veins studied here are the result of mineral growth within fluid‐filled fractures. The mode of fracturing may be deduced from the crystal shape and vein microtextures such as median lines and solid inclusion bands (Bons et al., 2012). The syntaxial veins are interpreted as mineralized mode I fractures possibly associated with extensional faulting. Their characteristic growth competition, the presence of a continuous median line, and the lack of solid inclusion bands suggest onefold crack and sealing (Bons et al., 2012; Fisher & Brantley, 1992; Ramsay, 1980). This implies that the rate of mineral growth approximated the rate of fracture opening (Fisher & Brantley, 1992; Hilgers et al., 2001). Extensional veining at Sites U1439–U1441 fits well into the concept of an extensional forearc regime where normal faults, grabens, and half‐grabens (Christeson et al., 2016; Robertson et al., 2018) related to slab rollback and trench retreat are observed (Faccenna et al., 2018; Kurz et al., 2019). In accordance with this interpretation, the Izu‐Bonin rear arc Site U1438 lacks syntaxial veins as well as large‐scale extensional structures (Arculus, Ishizuka, Bogus, Gurnis, et al., 2015; Arculus, Ishizuka, Bogus, & the Expedition 351 Scientists, 2015).

#### 5.1.2 Blocky Veins

Irregular branching blocky vein networks enclosing host rock fragments point to the mineralization of hydrofractures that formed due to fluid overpressure (Bons et al., 2012; Jébrak, 1997; Tartarotti & Pasquaré, 2003). Crosscutting relationships and the incorporation of antitaxial vein fragments (ASB‐4) into blocky veins (ASB‐5) (Figure 4g) indicate repeated fracturing. Alternatively, recrystallization might have altered original vein microtextures and resulted in blocky crystal shapes (Bons et al., 2012; Williams & Urai, 1989).

Since syntaxial and blocky veins show a textural continuum and distinguishing features such as growth competition and median lines may be incomplete, rates of fracture opening to fracture sealing must have varied continually. Therefore, the typical characteristics of syntaxial veins are less evolved where mineral growth did not keep pace with fracture opening, resulting in mineral growth within fluid‐filled space (Bons et al., 2012; Fisher & Brantley, 1992; Hilgers et al., 2001). Pure growth within fluid‐filled space is unambiguously expressed by blocky veins in which ongoing nucleation and mineral growth without preferred orientation (Bons et al., 2012) resulted in irregularly arranged blocky calcites. Euhedral, early‐stage zeolites on the vein margins give further evidence of mineral growth within fluid‐filled fractures.

During their growth, calcites in veins from the Izu‐Bonin forearc developed Mn‐activated (Fairchild, 1983) growth zonations consisting of one to four luminescent zones including the grain boundary (Figure 4h). Growth zonations are commonly interpreted as a result of changing fluid compositions associated with an open system, varying redox, temperature or pressure conditions, alternating growth rates, or closed system‐related geochemical self‐organization out of equilibrium (Dromgoole & Walter, 1990; Meyer, 1984; Ortoleva et al., 1987; Reeder et al., 1990; Shore & Fowler, 1996; Wang & Merino, 1992).

Changing fluid compositions may originate from mixing of seawater with hydrothermal fluid and/or different rates of fluid‐rock interaction. The fine and repetitive character of the more complex oscillatory growth zonations consisting of more than two zones (e.g., BON‐14 and FAB‐16/19; Figure 4h), however, argues against changing fluid compositions and points to geochemical self‐organization. During this process calcites autonomously develop growth zonations without extrinsic control (Ortoleva et al., 1987). Therefore, geochemical self‐organization occurs in a closed system out of equilibrium (Reeder et al., 1990). This implies that the stable and clumped isotopic compositions of calcites showing complex growth zonations may not reflect the composition of the fluid from which they precipitated.

More simple growth zonations solely consisting of a nonluminescent core and a thin luminescent grain boundary (e.g., FAB‐7/9/12/17/18/22; growth zonation is similar to the elongate‐blocky calcite in Figure 4c), however, may record precipitation in equilibrium with the fluid. In this case the nonluminescent core may represent relatively pure calcite (Habermann et al., 1998). The luminescent grain boundary may reflect Mn adsorption on the calcite surface (Franklin & Morse, 1983; McBride, 1979) at the end of crystal growth when the Mn/Ca ratio of fluid was increased due to preferred Ca incorporation into the core.

#### 5.1.3 Antitaxial Veins

The origin of antitaxial veining and its mode of fluid supply are still under debate (Bons et al., 2012). In previous studies on natural antitaxial fibrous veins, crack and sealing (Ramsay, 1980) and microbe‐induced calcite fiber growth (Bons et al., 2009; Bons & Montenari, 2005) were proposed as potential formation processes. However, the majority of previous studies agrees on fracture‐independent veining solely due to the crystallization pressure of fibrous mineral growth involving considerable diffusive fluid supply (e.g., Elburg et al., 2002; Meng et al., 2019; Wiltschko & Morse, 2001). This hypothesis is corroborated by laboratory experiments (e.g., Bons & Jessell, 1997; Means & Li, 2001; Taber, 1916) and numerical simulations (e.g., Hilgers et al., 2001), in which antitaxial fibrous microtextures were successfully generated due to the crystallization pressure of fibers.

These natural and synthetic antitaxial veins from the literature show microtextural characteristics that are strongly reminiscent of the antitaxial veins studied here. We therefore suggest that fluid diffusion and the crystallization pressure of calcite fibers induced antitaxial veining in the Izu‐Bonin rear arc and subordinately in the forearc. This is in accordance with the conspicuous high number of antitaxial veins in the rear arc where faults representing efficient advective fluid channels were not observed and a sedimentary cover reducing the permeability of the oceanic crust was present during the Eocene (Arculus, Ishizuka, Bogus, Gurnis, et al., 2015; Arculus, Ishizuka, Bogus, & the Expedition 351 Scientists, 2015; Coogan & Gillis, 2018; Fisher, 1998; Pearson & Lister, 1973). This geological setting limited the advective fluid transport in the rear arc and provided conducive conditions for antitaxial veining.

The forearc, in contrast, is characterized by multiple normal faults (Christeson et al., 2016; Kurz et al., 2019; Reagan et al., 2015) that might have channelized advective fluid circulation, which in turn hampered antitaxial veining. Hence, blocky veins dominate the forearc, whereas well‐developed antitaxial veins are rare. As a consequence of crystallization pressure‐driven antitaxial veining, predominantly straight calcite fibers join corresponding markers along the parallel vein margins and indicate the vein opening trajectory (Bons et al., 2012; Durney & Ramsay, 1973; Ramsay & Huber, 1983).

#### 5.1.4 Transitional Veins

The textural continuum between syntaxial and antitaxial veins reflects varying rates of mineral growth to fracture opening (Bons et al., 2012; Fisher & Brantley, 1992; Hilgers et al., 2001). The highest rates are recorded where fracture opening approximated zero and antitaxial calcite fibers display extreme length‐width ratios (Bons et al., 2012; Hilgers et al., 2001). Transitional veins have length‐width ratios ~10 and thus imply reduced rates of mineral growth to fracture opening. Transitional veins occur predominantly in the Izu‐Bonin forearc Sites U1439–U1441, whereas the rear arc Site U1438 exposes a high number of purely antitaxial veins with length‐width ratios >100. We therefore suggest that the extensional regime in the Izu‐Bonin forearc (Christeson et al., 2016; Kurz et al., 2019) inhibited widespread antitaxial veining. Transitional veins may accordingly reflect antitaxial veining that was preceded, accompanied, or succeeded by extensional fracturing, whose path of propagation was predetermined by zones of weakness such as preexisting (antitaxial) veins.

#### 5.1.5 Micrite Veins

Micrite‐filled fractures with similar petrographic characteristics as the micrite veins studied in this investigation have been discovered in numerous drill cores recovered during IODP Expeditions and on land (e.g., Christie et al., 2001; Clerc et al., 2014; Quandt et al., 2018; Schroeder et al., 2015). Their origin and formation, however, is often not discussed in detail and remains vague. In studies focusing on the alteration of the oceanic crust, fluid‐mediated injection or infill of calcareous sediments into fractures or cavities, that is, neptunian dykes (e.g., Lehner, 1991, and references therein), is often suggested (Christie et al., 2001; Clerc et al., 2014; Schroeder et al., 2015).

Though the Izu‐Bonin forearc is characterized by a long sedimentary hiatus until 35 Ma, bypassing sediment (Robertson et al., 2018) was probably captured by fractures connected to the seafloor. Positive δ^13^C values preclude any significant contribution from organic and mantle‐derived carbon. Instead, stable isotopic compositions are compatible with the precipitation from seawater and mobilized sediment. Therefore, the micrite‐filled veins may represent neptunian dykes. This explains the observed lamination and the increased occurrence in the upper levels of the drill cores, whereas the deeper rear arc drill cores lack any micritic component (Figure 2). Deeper micrites lacking lamination as well as noncarbonate phases and being spatially associated with blocky and fibrous calcites, by contrast, may have been precipitated from seawater similarly to blocky calcites.

### 5.2 Geochemical Composition of Circulating Fluids and Temperature of Calcite Veining

#### 5.2.1 U1440 and U1441 (FAB, forearc)

FAB‐hosted vein calcites of all types from Sites U1440 and U1441 show geochemical signatures indicative of unmodified seawater. Their stable isotopic compositions fall within or close to the field of global Eocene deep‐sea carbonates (Zachos et al., 2001), and parental fluid δ^18^O compositions based on Δ_47_ values are in accordance with seawater to seawater‐like fluids. Intersections of ^87^Sr/^86^Sr ratios of vein calcites with the ^87^Sr/^86^Sr seawater curve (McArthur et al., 2001) cannot exclude precipitation from seawater but may also be the result of mixing between Oligocene or younger seawater and mantle‐derived ^87^Sr/^86^Sr.

Furthermore, REE+Y distribution patterns exhibit almost consistently the typical negative Ce and the positive Y anomalies of seawater (Zhang & Nozaki, 1996), whereas pronounced positive Eu anomalies indicative of hydrothermal fluid involvement (Bau & Dulski, 1999) are absent. Positive Eu anomalies are caused by the fractionation of Eu^2+^ from the trivalent REE+Y and give rough information on the fluid temperature evolution prior to mineral precipitation (Bau et al., 2010). In contrast to Eu^+3^ and the neighboring trivalent REEs, Eu^2+^ is stable at temperatures ≥250 °C (Sverjensky, 1984). The resulting fractionation is only weakly dependent on pressure, pH, and the alteration of plagioclase (Allen & Seyfried, 2005; Bau, 1991; Danielson et al., 1992; Sverjensky, 1984). Cooling of a ≥250 °C hot hydrothermal fluid does not alter the positive Eu anomaly, and mixing with cool seawater only diminishes the Eu anomaly (Bau et al., 2010).

Consistently negative Ce/Ce*_PAAS_ and the corresponding absence of pronounced positive Eu/Eu*_PAAS_ point to well‐oxygenated seawater that was not modified by fluid‐rock interactions as indicated by Y/Ho ratios mostly >40. These characteristics are based on the redox‐sensitive fractionation of Ce^+4^ from the trivalent REE and the preferred sorption of Ho relative to Y on particulate matter in marine environments, respectively (Bau et al., 1997; Bau & Dulski, 1999; Möller, 2002; Nozaki et al., 1997).

Stable oxygen and clumped isotope geothermometry uniformly reveal temperatures <30 °C. This low‐temperature calcite formation agrees with the locally associated occurrence of the low‐temperature zeolite mineral phillipsite, which is stable below 100 °C and represents an alteration product of basaltic glass (Chipera & Apps, 2001). The decrease of δ^18^O values equivalent to increasing formation temperatures with increasing depth below seafloor (Figure 7) may be the result of a geothermal gradient. In a few cases, repeated δ^18^O analyses of individual blocky calcites reveal varying δ^18^O values (Table 1). Together with the occurrence of complex growth zonations (e.g., FAB‐16/19), this probably reflects variable physicochemical conditions during calcite growth (e.g., growth rate and temperature), changing fluid compositions due to fluid mixing or global changes in seawater chemistry over time, or geochemical self‐organization. Most blocky calcites (e.g., FAB‐7/9/12/17/18), however, show consistent δ^18^O values and simple growth zonations indicative of equilibrium precipitation from seawater.

Based on these geochemical interpretations and the predominance of blocky veins associated with brecciated host rocks, the Izu‐Bonin forearc at Sites U1440 and U1441 must have experienced extensive hydrofracturing accompanied by minor extensional fracturing indicated by rare syntaxial veins. Hydrofractures propagated upward to the seafloor where the advective fluid circulation system was recharged (Alt, 1995). Argillaceous vein selvedges formed prior to blocky calcites as the result of fluid‐rock interaction. They formed in equilibrium with the prevailing physicochemical conditions and reduced the host rock area in fractures exposed to circulating fluids. Therefore, the vein selvedges possibly attenuated further fluid‐rock interaction.

#### 5.2.2 U1439 (Boninite, Forearc)

Boninite‐hosted vein calcites from Site U1439 show similar isotopic signatures as FAB‐hosted vein calcites, implying low‐temperature precipitation under similar conditions. Stable oxygen and clumped isotopic compositions indicate precipitation temperatures <30 °C and show a correlation with depth following a geothermal gradient. Further similarities include stable carbon and oxygen isotopic compositions overlapping with the global Eocene deep‐sea carbonate field (Zachos et al., 2001), parental fluid δ^18^O compositions within the seawater range, and intersections of sample ^87^Sr/^86^Sr ratios with the Sr isotope seawater curve (McArthur et al., 2001).

However, some vein calcites (BON‐10/11/19/20) have ^87^Sr/^86^Sr ratios slightly below the ^87^Sr/^86^Sr seawater curve, pointing to the involvement of mantle‐derived Sr. Trace element characteristics show further differences between vein calcites from the different forearc sites. Boninite‐hosted vein calcites have less pronounced negative or even positive Ce/Ce*_PAAS_, larger Eu/Eu*_PAAS_, and lower Y/Ho ratios than have FAB‐hosted vein calcites, indicating a reducing environment and more intense fluid‐rock interaction at Site U1439. These modified seawater signatures may be related to the lack of selvedges possibly representing a protection from fluid‐rock interaction, higher extent of fluid‐rock interaction as indicated by pervasively altered boninite compared to localized alteration patterns of FAB, and/or higher proportion of antitaxial and transitional veins, whose diffusion‐fed growth facilitated host rock leaching. As a consequence of fluid‐rock interaction, the alkalinity in the fluid was increased, leading to calcite supersaturation (Sample et al., 2017); the Y/Ho ratios of the fluid were reduced toward the host rock value (~26) (Shervais et al., 2019); and the fluid δ^18^O was slightly lowered relative to seawater (Gregory & Taylor, 1981; Muehlenbachs & Clayton, 1976). The latter is indicated by clumped isotopes, which yield slightly lower parental fluid δ^18^O compositions than FAB‐hosted vein calcites. An increase in fluid alkalinity is probably accompanied by mobilization of Sr from the host rock resulting in an ^87^Sr/^86^Sr decrease of ambient seawater.

Both Mn‐controlled blocky calcite growth zonation (e.g., BON‐14) and varying Ce/Ce*_PAAS_ probably indicate redox‐sensitive changes during mineral growth. Moreover, varying conditions during calcite growth are suggested by variable stable isotopic compositions of individual vein samples (BON‐4/22/26) also observed among FAB‐hosted vein calcites (Table 1). The textural continuum between syntaxial and antitaxial veins (i.e., transitional veining) is supported by stable isotopic and trace element characteristics in Figures 6 and 12 in which transitional vein calcites show intermediate compositions between syntaxial and antitaxial vein calcites.

Similarly to the FAB Sites U1440 and U1441, hydrofracturing, advective fluid flow, and low‐temperature calcite precipitation modified the boninitic crust at Site U1439. Elemental and isotopic tracers, however, indicate that the circulating seawater underwent more intense modification due to fluid‐rock interactions than that at Sites U1440 and U1441.

#### 5.2.3 U1438 (FAB‐Like, Rear Arc)

In contrast to the vein calcites from the Izu‐Bonin forearc, the vein calcites from the rear arc show a considerable range of δ^18^O, δ^13^C, Δ_47_, and ^87^Sr/^86^Sr isotopic compositions, pointing to the formation under variable physicochemical conditions. On the one hand, this range includes seawater‐like ^87^Sr/^86^Sr ratios and parental fluid δ^18^O values within the range of seawater (ASB‐4/9). Together with corresponding seawater‐like average Y/Ho ratios >36, Eu/Eu*_PAAS_ ≤1.3, and Ce/Ce*_PAAS_ <1 (ASB‐4/11), precipitation from slightly modified seawater similar to the vein calcites from the forearc is suggested. On the other hand, the range of isotopic compositions also involves the lowest δ^18^O, parental fluid δ^18^O, δ^13^C, and ^87^Sr/^86^Sr values, as well as the highest formation temperatures and Eu/Eu*_PAAS_ of all samples (ASB‐1/3/5/10/12).

Very low parental δ^18^O fluid values relative to seawater (−3.3‰ to −9.7‰ VSMOW, ASB‐1/5) are likely the result of low‐T fluid‐rock interaction (Gregory & Taylor, 1981; Muehlenbachs & Clayton, 1976). Low‐T seawater‐basalt interaction results in ^18^O enrichment of the basalt and ^18^O depletion in the fluid (Gregory & Taylor, 1981; Muehlenbachs & Clayton, 1976), from which the calcites precipitated. The corresponding samples have ^87^Sr/^86^Sr ratios below Eocene seawater values. Therefore, clumped and Sr isotopic compositions consistently indicate that vein calcites from the rear arc precipitated from seawater that was modified by fluid‐rock interaction. As a result of this ^18^O depletion in the parental fluid, stable oxygen isotope thermometry‐based precipitation temperatures are overestimated. We therefore suggest that precipitation temperatures of vein calcites from the rear arc did not exceed the *T*_Δ47_ of 74 ± 12 °C.

Compared to veining in the forearc, vein calcites from the rear arc show higher formation temperatures, indicate more altered seawater compositions, and are more often classified as antitaxial veins. These features can be related to the local geological setting of the rear arc. Veins from Izu‐Bonin rear arc Site U1438 were recovered from significantly greater depth between 1,460 and 1,600 m below seafloor compared to the <500 m depth of the veins from the forearc (Figure 2). These differences may explain higher precipitation temperatures due to increasing temperature with increasing depth. Furthermore, the lack of major fault structures in the rear arc and the presence of a low‐permeability sedimentary cover during the Eocene (Arculus, Ishizuka, Bogus, Gurnis, et al., 2015; Arculus, Ishizuka, Bogus, & the Expedition 351 Scientists, 2015) resulted in limited advective fluid circulation (Coogan & Gillis, 2018; Fisher, 1998; Pearson & Lister, 1973) and provoked fluid diffusion instead. This facilitated the growth of diffusion‐fed and fault‐independent crystallization pressure‐driven antitaxial veins (Elburg et al., 2002; Means & Li, 2001; Meng et al., 2019; Taber, 1916; Wiltschko & Morse, 2001). Diffusion in turn intensified fluid‐rock interaction and resulted in varying degrees of seawater modification.

### 5.3 Chronology of Veining

Calcite that forms in the marine environment under equilibrium conditions records the ^87^Sr/^86^Sr ratio of the fluid from which it precipitates without fractionation (Kawahata et al., 2001). Therefore, intersections of Sr isotopic compositions of vein calcites with the Sr isotope seawater curve (McArthur et al., 2001) enable the relative dating of calcite precipitation (Hart & Staudigel, 1978) and fracturing in case of syntaxial crack and sealing veins due to simultaneous fracturing and calcite precipitation (Ramsay, 1980; Roberts & Walker, 2016). The potential involvement of mantle‐derived strontium with ^87^Sr/^86^Sr ratios ~0.70300 (Albarède et al., 1981) lowers the ^87^Sr/^86^Sr ratio of the sample. Due to increasing seawater ^87^Sr/^86^Sr ratios since ~40 Ma (McArthur et al., 2001), a contribution of mantle strontium would increase the apparent age of a sample, and thus, estimated ages represent maximum ages. For instance, a sample with an ^87^Sr/^86^Sr ratio of 0.70780 intersects the Sr isotope seawater curve at ~34 Ma. If this sample resulted from the mixture of seawater and basaltic ^87^Sr/^86^Sr (~0.70300), then a higher seawater ^87^Sr/^86^Sr ratio (>0.70780) equivalent to a younger intersection age would be required in order to result in the mixed sample ^87^Sr/^86^Sr ratio.

Except for vein calcites from the rear arc (ASB‐1/4/5/8/9/14) and a few boninite‐hosted vein calcites from the forearc (BON‐10/11/19/20), vein calcites independently of the type intersect the Sr isotope seawater curve between ~52 and ~22 Ma. Due to the sigmoidal shape of the Sr isotope seawater curve, particularly boninite‐hosted vein calcites show multiple intersections. Vein calcites from the rear arc and boninite‐hosted vein calcites are also characterized by precipitation from variably modified seawater due to fluid‐rock interaction. This probably involved the exchange of Sr between basalt and seawater. We therefore conclude that vein calcites from the rear arc and boninite‐hosted vein calcites cannot be dated reliably using this method. Thus, these vein calcites may have formed between respective host rock emplacement (≤52 Ma) and today.

In contrast, FAB‐hosted vein calcites show the most pristine seawater signatures and, in most cases, single intersections with the Sr isotope seawater curve (FAB‐4/10/18/22). Their stable and clumped isotopic compositions indicate precipitation from seawater, and REE+Y distribution patterns show the typical features of seawater (e.g., negative Ce and positive Y anomalies). FAB‐hosted vein calcites also define the upper parts of the δ^18^O‐^87^Sr/^86^Sr‐depth correlations in Figures 7 and 10, which indicate the most seawater‐like compositions. CL petrography shows that the corresponding samples (FAB‐7/18/22) are characterized by a nonluminescent core and a thin luminescent grain boundary. Based on this observation, equilibrium precipitation from fluids with constant composition is inferred. We therefore suggest that vein calcite precipitation within FAB may be dated by using the Sr isotope seawater curve. Thus, most vein calcites hosted in FAB and showing single intersections with the Sr isotope seawater curve point to calcite precipitation between ~35 and ~33 Ma.

A single calcite vein (FAB‐18) hosted in a FAB talus (Figure 2) intersects the Sr isotope seawater curve at ~22 Ma (Figure 9). The FAB talus probably formed by erosion of extensional fault scarps in Miocene to Pliocene times (Robertson et al., 2018). The vein is intact and therefore appears to have formed after talus formation. This is in agreement with its intersection age, which represents a maximum precipitation age.

The precipitation ages of FAB‐hosted vein calcites fall mainly within an interval of 25 Myr after crust formation (~52 Ma). This represents the typical temporal framework during which >80% of secondary mineralization is completed (e.g., Coogan & Gillis, 2018, and references therein; Hart et al., 1994; Hart & Staudigel, 1978; Quandt, Micheuz, Kurz, Kluge, et al., 2019; Richardson et al., 1980; Staudigel & Hart, 1985; Staudigel et al., 1981). The precipitation ages also overlap with the onset of pelagic carbonate deposition within extensional graben and half‐graben basins along the Izu‐Bonin forearc (Robertson et al., 2018). Thus, veining of FAB is possibly related to Pacific slab rollback and trench retreat from ~35 Ma onward, which triggered extension of the upper plate (Faccenna et al., 2018; Kurz et al., 2019).

### 5.4 Quantification of the Extent of Fluid‐Rock Interaction

The geochemical compositions of vein calcites indicate that they precipitated from variable fluid compositions ranging from pristine seawater (FAB‐hosted vein calcites) to weakly and significantly modified seawater compositions due to fluid‐rock interaction (rear arc and boninite‐hosted vein calcites). This fluid‐rock interaction will be quantified in the following using simple binary mixing calculations. These calculations are based on a pristine seawater end‐member with a fixed ^87^Sr/^86^Sr ratio for a given time and a basaltic end‐member with a fixed ^87^Sr/^86^Sr ratio. This means that a precipitation age equivalent to the fixed seawater ^87^Sr/^86^Sr ratio is assumed and the necessary fraction of host rock ^87^Sr/^86^Sr is calculated in order to yield the sample ^87^Sr/^86^Sr ratio.

Since veining may have occurred before, contemporaneously with, or after pervasive host rock alteration, two approaches are presented. The first approach assumes pristine seawater reaction with a fresh basaltic host rock, whereas the second approach supposes pristine seawater reaction with an altered basaltic host rock. The latter is supported by some calcite veins that crosscut altered phenocrystals and zeolite veins, which represent alteration products.

Calculations were performed using the following equation (^87^Sr/^86^Sr)_Mixture_ = [Sr_Seawater_ × (^87^Sr/^86^Sr)_Seawater_ × (1 − *f*_Host rock_) + Sr_Host rock_ × (^87^Sr/^86^Sr)_Host rock_ × *f*_Host rock_]/[Sr_Seawater_ × (1 − *f*_Host rock_) + Sr_Host rock_ × *f*_Host rock_], where (^87^Sr/^86^Sr) is the Sr isotope composition of the respective component, Sr is the strontium concentration of the respective component, and *f* is the weight fraction of the respective component in the mixture (Faure & Mensing, 2005). The respective components are indicated by “Seawater,” “Host rock,” and “Mixture” indices.

For both approaches a range of pristine seawater ^87^Sr/^86^Sr ratios corresponding to ~34 Ma (0.7078), ~22 Ma (0.70836), and ~5 Ma (0.70903) (McArthur et al., 2001) were used. The two older ages correspond to the assumed precipitation ages of FAB‐hosted seawater‐derived vein calcites. For fresh and altered basaltic end‐members, ^87^Sr/^86^Sr ratios of 0.7032 and 0.7050 were used, respectively (Reagan et al., 2010; Yogodzinski et al., 2018). Between the Eocene and today, the Sr concentration of seawater has mainly decreased from ~14 to ~7.8 ppm (Coogan, 2009). Based on Coogan (2009), Sr concentrations of 12, 10, and 8 ppm, representative for seawater at 34, 22, and 5 Ma, respectively, were used for the calculations. FAB and boninites have average Sr concentrations of ~100 ppm (Reagan et al., 2010). During seawater‐induced alteration, Sr is mobilized but may be incorporated into alteration phases balancing the whole rock Sr concentration (Hart, 1971; Humphris & Thompson, 1978; Menzies & Seyfried, 1979). Therefore, 100 ppm Sr was used for both approaches. Mixed ^87^Sr/^86^Sr ratios were calculated for 1%, 5%, 10%, 20%, 30%, and 40% fractions of basaltic ^87^Sr/^86^Sr.

**Figure 13:**
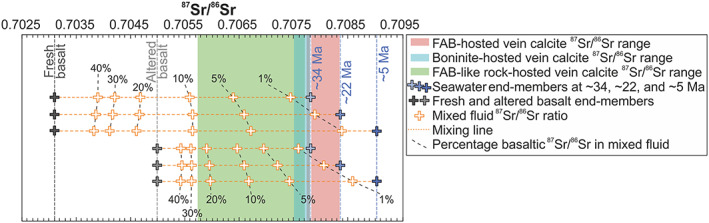
Simple binary mixing calculations (Faure & Mensing, 2005) between a basalt and a seawater end‐member. Calculations are based on ^87^Sr/^86^Sr ratios and Sr concentrations of seawater at ~34 Ma (0.70780, 12 ppm), ~22 Ma (0.70836, 10 ppm), and ~5 Ma (0.70903, 8 ppm) (Coogan, 2009; McArthur et al., 2001). These seawater compositions reacted with fresh (0.70320, 100 ppm) and altered basalt (0.70500, 100 ppm) (Reagan et al., 2010; Yogodzinski et al., 2018). Percentages give the basaltic ^87^Sr/^86^Sr fraction within the mixed fluid relative to the seawater ^87^Sr/^86^Sr fraction. For instance, the lowest ^87^Sr/^86^Sr ratio of the sample suite (0.70574 ± 0.00001, vein calcite from the rear arc) may have resulted from mixing of ~90% seawater with ~10% fresh host rock. If the host rock was altered before veining, 20% to 30% host rock‐derived ^87^Sr/^86^Sr was necessary. The remaining rear arc veins and boninite‐hosted veins require host rock fractions <10%.

Figure 13 shows the results of the mixing calculations. Due to its high Sr concentration, the basaltic end‐member controls the ^87^Sr/^86^Sr ratio of the mixed fluid. The different time‐dependent seawater ^87^Sr/^86^Sr ratios have an impact on the seawater‐dominated mixtures but do not significantly affect host rock‐dominated mixtures. The lowest ^87^Sr/^86^Sr ratio of the sample suite recorded by a vein calcite from the rear arc (ASB‐5) may have resulted from mixing of ~90% seawater with ~10% fresh host rock. If the host rock was altered before veining, 20% to 30% host rock‐derived ^87^Sr/^86^Sr was necessary. The remaining rear arc vein calcites (ASB‐1/4/8/9/14) have higher ^87^Sr/^86^Sr ratios, which require host rock fractions <10%. The basaltic ^87^Sr/^86^Sr fractions for boninite‐hosted vein calcites depend on the time of precipitation but are generally <5%. In summary, most rear arc and boninite‐hosted vein calcites precipitated from seawater‐dominated fluids with <10% basaltic ^87^Sr/^86^Sr contribution.

## 6 Conclusion

Geochemical and microtextural observations indicate that calcite veining in the Izu‐Bonin forearc and rear arc is dominated by different processes under diverse physicochemical conditions. These differences can be related to the local geological settings. In the forearc, blocky calcite veins predominate over syntaxial and antitaxial calcite veins and indicate intense hydrofracturing and advective fluid flow. Major faults and the lack of a sedimentary cover (Christeson et al., 2016; Robertson et al., 2018) facilitated low‐T advective fluid flow through the forearc crust. The rear arc, in contrast, shows approximately equal proportions of blocky and antitaxial calcite veins. The lack of major faults and the early presence of a sedimentary cover (Arculus, Ishizuka, Bogus, Gurnis, et al., 2015; Arculus, Ishizuka, Bogus, & the Expedition 351 Scientists, 2015) potentially hampered advective fluid circulation and provided conducive conditions for diffusion‐fed and crystallization pressure‐driven antitaxial veining. Therefore, antitaxial veins may constitute an efficient veining mechanism within oceanic crust where faults and other advective fluid paths are absent or have previously been sealed by secondary mineralization and/or sedimentation.

As a consequence of these dissimilar geological settings and different vein types in the forearc and rear arc, vein calcites reveal varying geochemical signatures indicative of distinct geological and physicochemical processes. The sample suite defines a mixing curve from pristine seawater to variably modified seawater. The most pristine geochemical seawater signatures are recorded by FAB‐hosted vein calcites whose oxygen and clumped isotopic compositions reveal precipitation temperatures <30 °C. Their REE+Y distribution patterns display the typical characteristics of seawater (negative Ce and positive Y anomalies), and clumped isotopes indicate parental fluid δ^18^O compositions in the range of seawater. ^87^Sr/^86^Sr ratios of FAB‐hosted vein calcites intersecting the Sr isotope seawater curve (McArthur et al., 2001) between ~35 and ~ 33 Ma and at ~22 Ma are therefore interpreted as precipitation ages. This time interval overlaps with Pacific slab rollback and Izu‐Bonin trench retreat (Faccenna et al., 2018), which might have triggered upper plate extension and fracturing (Kurz et al., 2019).

Oxygen and clumped isotopic compositions of boninite‐hosted vein calcites point to precipitation temperatures <30 °C similar to FAB‐hosted vein calcites. However, boninite‐hosted vein calcites show lower ^87^Sr/^86^Sr and parental fluid δ^18^O compositions than FAB‐hosted vein calcites indicating weak, low‐temperature fluid‐rock interaction. This is in agreement with reduced Y anomalies and increased Eu anomalies relative to seawater. Simple binary mixing calculations yield <5% basaltic ^87^Sr/^86^Sr contribution presuming vein calcite precipitation at ~34, ~22, and ~5 Ma.

A few blocky vein calcites from the forearc developed Mn‐controlled oscillatory growth zonations or show varying Ce anomalies along line profiles through the veins. These observations indicate varying physicochemical conditions during calcite growth (e.g., varying growth rates, temperatures, redox conditions, and/or fluid compositions) or geochemical self‐organization. The latter implies crystal growth within a closed system out of equilibrium (e.g., Ortoleva et al., 1987; Reeder et al., 1990). The geochemical interpretation of such samples has to be treated with care as they might be the result of disequilibrium precipitation.

Vein calcites from the rear arc show variable isotopic and trace element signatures due to varying degrees of fluid‐rock interaction. Vein calcites with ^87^Sr/^86^Sr ratios slightly below the seawater curve, parental fluid δ^18^O compositions within the range of seawater, and seawater‐like REE+Y patterns are also observed among boninite‐hosted vein calcites in the forearc and indicate precipitation from slightly modified seawater due to weak fluid‐rock interaction. More modified ^87^Sr/^86^Sr ratios considerably below the seawater curve, very low parental fluid δ^18^O compositions, and elevated precipitation temperatures up to 74 ± 12 °C are distinct characteristics of vein calcites from the rear arc. These geochemical characteristics are probably related to the greater depth from which the veins were recovered and low‐temperature fluid‐rock interaction during which up to 20–30% basaltic ^87^Sr/^86^Sr was contributed to the fluid mixture from which the calcites precipitated.

This study shows the benefits and additional insights provided by the integration of vein microtextures into a geochemical study and demonstrates how the interplay of the local geological setting, mode of fluid flow and fracturing, and the physicochemical environment determines the geochemical composition of vein calcites and their microtextures.

## References

[ggge22131-bib-0001] Albarède, F. , Michard, A. , Minster, J. F. , & Michard, G. (1981). ^87^Sr/^86^Sr ratios in hydrothermal waters and deposits from the East Pacific Rise at 21°N. Earth and Planetary Science Letters, 55(2), 229–236. 10.1016/0012-821X(81)90102-3

[ggge22131-bib-0002] Albers, E. , Bach, W. , Klein, F. , Menzies, C. D. , Lucassen, F. , & Teagle, D. A. (2019). Fluid–rock interactions in the shallow Mariana forearc: Carbon cycling and redox conditions. Solid Earth, 10(3), 907–930. 10.5194/se-10-907-2019

[ggge22131-bib-0003] Allen, D. E. , & Seyfried, W. E. Jr. (2005). REE controls in ultramafic hosted MOR hydrothermal systems: An experimental study at elevated temperature and pressure. Geochimica et Cosmochimica Acta, 69(3), 675–683. 10.1016/j.gca.2004.07.016

[ggge22131-bib-0004] Alt, J. C. (1995). Subseafloor processes in mid‐ocean ridge hydrothermal systems In Humphris S. E., Zierenberg R. A., Mullineaux L. S., & Thomson R. E. (Eds.), Seafloor hydrothermal systems: Physical, chemical, biological, and geological interactions, (Vol. 91, pp. 85–114). Washington, DC: AGU Monograph 10.1029/GM091

[ggge22131-bib-0005] Alt, J. C. , & Teagle, D. A. (2003). Hydrothermal alteration of upper oceanic crust formed at a fast‐spreading ridge: Mineral, chemical, and isotopic evidence from ODP Site 801. Chemical Geology, 201(3‐4), 191–211. 10.1016/S0009-2541(03)00201-8

[ggge22131-bib-0006] Alt, J. C. , Teagle, D. A. , Brewer, T. , Shanks, W. C. , & Halliday, A. (1998). Alteration and mineralization of an oceanic forearc and the ophiolite‐ocean crust analogy. Journal of Geophysical Research ‐ Solid Earth, 103(B6), 12,365–12,380. 10.1029/98JB00598

[ggge22131-bib-0007] Arculus, R. J. , Ishizuka, O. , Bogus, K. , & the Expedition 351 Scientists (2015). Izu‐Bonin‐Mariana arc origins, Proceedings of the International Ocean Discovery Program, Expedition 351 TX (International Ocean Discovery Program): College Station 10.14379/iodp.proc.351.2015

[ggge22131-bib-0008] Arculus, R. J. , Ishizuka, O. , Bogus, K. A. , Gurnis, M. , Hickey‐Vargas, R. , Aljahdali, M. H. , Bandini‐Maeder, A. N. , Barth, A. P. , Brandl, P. A. , Drab, L. , do Monte Guerra, R. , Hamada, M. , Jiang, F. , Kanayama, K. , Kender, S. , Kusano, Y. , Li, H. , Loudin, L. C. , Maffione, M. , Marsaglia, K. M. , McCarthy, A. , Meffre, S. , Morris, A. , Neuhaus, M. , Savov, I. P. , Sena, C. , Tepley, F. J. III , van der Land, C. , Yogodzinski, G. M. , & Zhang, Z. (2015). A record of spontaneous subduction initiation in the Izu–Bonin–Mariana arc. Nature Geoscience, 8(9), 728 10.1038/ngeo2515

[ggge22131-bib-0009] Bau, M. (1991). Rare‐earth element mobility during hydrothermal and metamorphic fluid‐rock interaction and the significance of the oxidation state of europium. Chemical Geology, 93(3‐4), 219–230. 10.1016/0009-2541(91)90115-8

[ggge22131-bib-0010] Bau, M. , Balan, S. , Schmidt, K. , & Koschinsky, A. (2010). Rare earth elements in mussel shells of the Mytilidae family as tracers for hidden and fossil high‐temperature hydrothermal systems. Earth and Planetary Science Letters, 299(3‐4), 310–316. 10.1016/j.epsl.2010.09.011

[ggge22131-bib-0011] Bau, M. , & Dulski, P. (1999). Comparing yttrium and rare earths in hydrothermal fluids from the Mid‐Atlantic Ridge: Implications for Y and REE behaviour during near‐vent mixing and for the Y/Ho ratio of Proterozoic seawater. Chemical Geology, 155(1‐2), 77–90. 10.1016/S0009-2541(98)00142-9

[ggge22131-bib-0012] Bau, M. , Möller, P. , & Dulski, P. (1997). Yttrium and lanthanides in eastern Mediterranean seawater and their fractionation during redox‐cycling. Marine Chemistry, 56(1‐2), 123–131. 10.1016/S0304-4203(96)00091-6

[ggge22131-bib-0013] Bernasconi, S. M. , Hu, B. , Wacker, U. , Fiebig, J. , Breitenbach, S. F. M. , & Rutz, T. (2013). Background effects on Faraday collectors in gas‐source mass spectrometry and implications for clumped isotope measurements. Rapid Communications in Mass Spectrometry, 27(5), 603–612. 10.1002/rcm.6490 23413219

[ggge22131-bib-0014] Bernasconi, S. M. , Müller, I. A. , Bergmann, K. D. , Breitenbach, S. F. , Fernandez, A. , Hodell, D. A. , Jaggi, M. , Meckler, A. N. , Millan, I. , & Ziegler, M. (2018). Reducing uncertainties in carbonate clumped isotope analysis through consistent carbonate‐based standardization. Geochemistry, Geophysics, Geosystems, 19(9), 2895–2914. 10.1029/2017GC007385 PMC6220777 30443200

[ggge22131-bib-0015] Bloomer, S. H. , Taylor, B. , MacLeod, C. J. , Stern, R. J. , Fryer, P. , Hawkins, J. W. , & Johnson, L. (1995). Early arc volcanism and the ophiolite problem: A perspective from drilling in the western Pacific. Active margins and marginal basins of the western Pacific, 88, 1–30. 10.1029/GM088p0001

[ggge22131-bib-0016] Bons, P. D. (2001). Development of crystal morphology during unitaxial growth in a progressively widening vein: I. The numerical model. Journal of Structural Geology, 23(6‐7), 865–872. 10.1016/S0191-8141(00)00159-0

[ggge22131-bib-0017] Bons, P. D. , Elburg, M. A. , & Gomez‐Rivas, E. (2012). A review of the formation of tectonic veins and their microstructures. Journal of Structural Geology, 43, 33–62. 10.1016/j.jsg.2012.07.005

[ggge22131-bib-0018] Bons, P. D. , & Jessell, M. W. (1997). Experimental simulation of the formation of fibrous veins by localised dissolution‐precipitation creep. Mineralogical Magazine, 61(404), 53–63. 10.1180/minmag.1997.061.404.06

[ggge22131-bib-0019] Bons, P. D. , & Montenari, M. (2005). The formation of antitaxial calcite veins with well‐developed fibres, Oppaminda Creek, South Australia. Journal of Structural Geology, 27(2), 231–248. 10.1016/j.jsg.2004.08.009

[ggge22131-bib-0020] Bons, P. D. , Montenari, M. , Bakker, R. J. , & Elburg, M. A. (2009). Potential evidence of fossilised Neoproterozoic deep life: SEM observations on calcite veins from Oppaminda Creek, Arkaroola, South Australia. International Journal of Earth Sciences, 98(2), 327–343. 10.1007/s00531-007-0245-4

[ggge22131-bib-0021] Brandl, P. A. , Hamada, M. , Arculus, R. J. , Johnson, K. , Marsaglia, K. M. , Savov, I. P. , Ishizuka, O. , & Li, H. (2017). The arc arises: The links between volcanic output, arc evolution and melt composition. Earth and Planetary Science Letters, 461, 73–84. 10.1016/j.epsl.2016.12.027

[ggge22131-bib-0022] Brandstätter, J. , Kurz, W. , Krenn, K. , & Micheuz, P. (2016). Fluid inclusion petrology and microthermometry of the Cocos Ridge hydrothermal system, IODP Expedition 344 (CRISP 2), Site U1414. Geochemistry, Geophysics, Geosystems, 17, 1419–1434. 10.1002/2015GC006212 PMC4985704 27570496

[ggge22131-bib-0023] Brandstätter, J. , Kurz, W. , Richoz, S. , Cooper, M. J. , & Teagle, D. A. (2018). The origin of carbonate veins within the sedimentary cover and igneous rocks of the Cocos Ridge: Results from IODP Hole U1414A. Geochemistry, Geophysics, Geosystems, 19(10), 3721–3738. 10.1029/2018GC007729 PMC6282762 30546270

[ggge22131-bib-0024] Brandstätter, J. , Kurz, W. , & Rogowitz, A. (2017). Microstructural analysis and calcite piezometry on hydrothermal veins: Insights into the deformation history of the Cocos Plate at Site U1414 (IODP Expedition 344). Tectonics, 36, 1562–1579. 10.1002/2017TC004490 29081570 PMC5637954

[ggge22131-bib-0025] Burgess, C. E. , Pearson, P. N. , Lear, C. H. , Morgans, H. E. , Handley, L. , Pancost, R. D. , & Schouten, S. (2008). Middle Eocene climate cyclicity in the southern Pacific: Implications for global ice volume. Geology, 36(8), 651–654. 10.1130/G24762A.1

[ggge22131-bib-0026] Chipera, S. J. , & Apps, J. A. (2001). Geochemical stability of natural zeolites. Reviews in Mineralogy and Geochemistry, 45(1), 117–161. 10.2138/rmg.2001.45.3

[ggge22131-bib-0027] Christeson, G. L. , Morgan, S. , Kodaira, S. , Yamashita, M. , Almeev, R. R. , Michibayashi, K. , Sakuyama, T. , Ferré, E. C. , & Kurz, W. (2016). Physical properties and seismic structure of Izu‐Bonin‐Mariana fore‐arc crust: Results from IODP Expedition 352 and comparison with oceanic crust. Geochemistry, Geophysics, Geosystems, 17, 4973–4991. 10.1002/2016GC006638

[ggge22131-bib-0028] Christie, D. M. , Pedersen, R. B. , Miller, and the Shipboard Scientific Party (2001). Leg 187 summary. Proceedings of the ODP, 187. College Station TX (Ocean Drilling Program), 1–49.

[ggge22131-bib-0029] Clerc, C. , Boulvais, P. , Lagabrielle, Y. , & De Saint Blanquat, M. (2014). Ophicalcites from the northern Pyrenean belt: A field, petrographic and stable isotope study. International Journal of Earth Sciences, 103(1), 141–163. 10.1007/s00531-013-0927-z

[ggge22131-bib-0030] Coogan, L. A. (2009). Altered oceanic crust as an inorganic record of paleoseawater Sr concentration. Geochemistry, Geophysics, Geosystems, 10(4), Q04001 10.1029/2008GC002341

[ggge22131-bib-0031] Coogan, L. A. , Daëron, M. , & Gillis, K. M. (2019). Seafloor weathering and the oxygen isotope ratio in seawater: Insight from whole‐rock δ^18^O and carbonate δ^18^O and Δ_47_ from the Troodos ophiolite. Earth and Planetary Science Letters, 508, 41–50. 10.1016/j.epsl.2018.12.014

[ggge22131-bib-0032] Coogan, L. A. , & Gillis, K. M. (2018). Low‐temperature alteration of the seafloor: Impacts on ocean chemistry. Annual Review of Earth and Planetary Sciences, 46, 21–45. 10.1146/annurev-earth-082517-010027

[ggge22131-bib-0033] Coplen, T. B. (2007). Calibration of the calcite‐water oxygen‐isotope geothermometer at Devils Hole, Nevada, a natural laboratory. Geochimica et Cosmochimica Acta, 71(16), 3948–3957. 10.1016/j.gca.2007.05.028

[ggge22131-bib-0034] Daëron, M. , Blamart, D. , Peral, M. , & Affek, H. P. (2016). Absolute isotopic abundance ratios and the accuracy of Δ_47_ measurements. Chemical Geology, 442, 83–96. 10.1016/j.chemgeo.2016.08.014

[ggge22131-bib-0035] Danielson, A. , Möller, P. , & Dulski, P. (1992). The europium anomalies in banded iron formations and the thermal history of the oceanic crust. Chemical Geology, 97(1‐2), 89–100. 10.1016/0009-2541(92)90137-T

[ggge22131-bib-0036] Dromgoole, E. L. , & Walter, L. M. (1990). Iron and manganese incorporation into calcite: Effects of growth kinetics, temperature and solution chemistry. Chemical Geology, 81(4), 311–336. 10.1016/0009-2541(90)90053-A

[ggge22131-bib-0037] Durney, D. W. , & Ramsay, J. G. (1973). Incremental strains measured by syntectonic crystal growths In de Jong K. A., & Scholten R. (Eds.), Gravity and tectonics, (pp. 67–96). New York: Wiley.

[ggge22131-bib-0038] Eiler, J. M. , Crawford, A. , Elliott, T. I. M. , Farley, K. A. , Valley, J. W. , & Stolper, E. M. (2000). Oxygen isotope geochemistry of oceanic‐arc lavas. Journal of Petrology, 41(2), 229–256. 10.1093/petrology/41.2.229

[ggge22131-bib-0039] Elburg, M. A. , Bons, P. D. , Foden, J. , & Passchier, C. W. (2002). The origin of fibrous veins: Constraints from geochemistry. Geological Society, London, Special Publications, 200(1), 103–118. 10.1144/GSL.SP.2001.200.01.07

[ggge22131-bib-0040] Epstein, S. , Buchsbaum, R. , Lowenstam, H. A. , & Urey, H. C. (1953). Revised carbonate‐water isotopic temperature scale. Geological Society of America Bulletin, 64(11), 1315–1326. 10.1130/0016-7606(1953)64[1315:RCITS]2.0.CO;2

[ggge22131-bib-0041] Faccenna, C. , Holt, A. F. , Becker, T. W. , Lallemand, S. , & Royden, L. H. (2018). Dynamics of the Ryukyu/Izu‐Bonin‐Marianas double subduction system. Tectonophysics, 746, 229–238. 10.1016/j.tecto.2017.08.011

[ggge22131-bib-0042] Fairchild, I. J. (1983). Chemical controls of cathodoluminescence of natural dolomites and calcites: New data and review. Sedimentology, 30(4), 579–583. 10.1111/j.1365-3091.1983.tb00695.x

[ggge22131-bib-0043] Faure, G. , & Mensing, T. M. (2005). Isotopes: Principles and applications, (third ed.). New York: Wiley.

[ggge22131-bib-0044] Fisher, A. T. (1998). Permeability within basaltic oceanic crust. Reviews of Geophysics, 36(2), 143–182. 10.1029/97RG02916

[ggge22131-bib-0045] Fisher, D. M. , & Brantley, S. L. (1992). Models of quartz overgrowth and vein formation: Deformation and episodic fluid flow in an ancient subduction zone. Journal of Geophysical Research ‐ Solid Earth, 97(B13), 20,043–20,061. 10.1029/92JB01582

[ggge22131-bib-0046] Franklin, M. L. , & Morse, J. W. (1983). The interaction of manganese (II) with the surface of calcite in dilute solutions and seawater. Marine Chemistry, 12(4), 241–254. 10.1016/0304-4203(83)90055-5

[ggge22131-bib-0047] Frezzotti, M. L. , Tecce, F. , & Casagli, A. (2012). Raman spectroscopy for fluid inclusion analysis. Journal of Geochemical Exploration, 112, 1–20. 10.1016/j.gexplo.2011.09.009

[ggge22131-bib-0048] Friedman, I. , & O'Neil, J. R. (1977). Compilation of stable isotope fractionation factors of geochemical interest. In M. Fleischer (Ed.), Data of geochemistry, 6th edition. Geochemical Survey Professional Paper, 440, KK1–KK12. 10.3133/pp440KK

[ggge22131-bib-0049] Ghosh, P. , Adkins, J. , Affek, H. , Balta, B. , Guo, W. , Schauble, E. A. , Schrag, D. , & Eiler, J. M. (2006). ^13^C‐^18^O bonds in carbonate minerals: a new kind of paleothermometer. Geochimica et Cosmochimica Acta, 70(6), 1439–1456. 10.1016/j.gca.2005.11.014

[ggge22131-bib-0050] Gregory, R. T. , & Taylor, H. P. Jr. (1981). An oxygen isotope profile in a section of Cretaceous oceanic crust, Samail Ophiolite, Oman: Evidence for δ^18^O buffering of the oceans by deep (>5 km) seawater‐hydrothermal circulation at mid‐ocean ridges. Journal of Geophysical Research ‐ Solid Earth, 86(B4), 2737–2755. 10.1029/JB086iB04p02737

[ggge22131-bib-0051] Habermann, D. , Neuser, R. D. , & Richter, D. K. (1998). Low limit of Mn^2+^‐activated cathodoluminescence of calcite: State of the art. Sedimentary Geology, 116(1‐2), 13–24. 10.1016/S0037-0738(97)00118-8

[ggge22131-bib-0052] Hart, S. R. (1971). K, Rb, Cs, Sr and Ba contents and Sr isotope ratios of ocean floor basalts. Philosophical Transactions of the Royal Society of London. Series A, Mathematical and Physical Sciences, 268(1192), 573–587. 10.1098/rsta.1971.0013

[ggge22131-bib-0053] Hart, S. R. , Blusztajn, J. , Dick, H. J. , & Lawrence, J. R. (1994). Fluid circulation in the oceanic crust: Contrast between volcanic and plutonic regimes. Journal of Geophysical Research ‐ Solid Earth, 99(B2), 3163–3173. 10.1029/93JB02035

[ggge22131-bib-0054] Hart, S. R. , & Staudigel, H. (1978). Oceanic crust: Age of hydrothermal alteration. Geophysical Research Letters, 5(12), 1009–1012. 10.1029/GL005i012p01009

[ggge22131-bib-0055] Hickey‐Vargas, R. , Yogodzinski, G. M. , Ishizuka, O. , McCarthy, A. , Bizimis, M. , Kusano, Y. , Savov, I. P. , & Arculus, R. (2018). Origin of depleted basalts during subduction initiation and early development of the Izu‐Bonin‐Mariana island arc: Evidence from IODP Expedition 351 Site U1438, Amami‐Sankaku basin. Geochimica et Cosmochimica Acta, 229, 85–111. 10.1016/j.gca.2018.03.007

[ggge22131-bib-0056] Hilgers, C. , Dilg‐Gruschinski, K. , & Urai, J. L. (2004). Microstructural evolution of syntaxial veins formed by advective flow. Geology, 32(3), 261–264. 10.1130/G20024.1

[ggge22131-bib-0057] Hilgers, C. , Koehn, D. , Bons, P. D. , & Urai, J. L. (2001). Development of crystal morphology during unitaxial growth in a progressively widening vein: II. Numerical simulations of the evolution of antitaxial fibrous veins. Journal of Structural Geology, 23(6‐7), 873–885. 10.1016/S0191-8141(00)00160-7

[ggge22131-bib-0058] Hilgers, C. , & Urai, J. L. (2002). Experimental study of syntaxial vein growth during lateral fluid flow in transmitted light: First results. Journal of Structural Geology, 24(6‐7), 1029–1043. 10.1016/S0191-8141(01)00089-X

[ggge22131-bib-0059] Hoefs, J. (2015). Stable isotope geochemistry. Springer‐Verlag, Switzerland, 2015, 1–389. 10.1007/978-3-319-19716-6

[ggge22131-bib-0060] Holt, A. F. , Royden, L. H. , Becker, T. W. , & Faccenna, C. (2018). Slab interactions in 3‐D subduction settings: The Philippine Sea Plate region. Earth and Planetary Science Letters, 489, 72–83. 10.1016/j.epsl.2018.02.024

[ggge22131-bib-0061] Humphris, S. E. , & Thompson, G. (1978). Trace element mobility during hydrothermal alteration of oceanic basalts. Geochimica et Cosmochimica Acta, 42(1), 127–136. 10.1016/0016-7037(78)90222-3

[ggge22131-bib-0062] Ishizuka, O. , Hickey‐Vargas, R. , Arculus, R. J. , Yogodzinski, G. M. , Savov, I. P. , Kusano, Y. , McCarthy, A. , Brandl, P. A. , & Sudo, M. (2018). Age of Izu–Bonin–Mariana arc basement. Earth and Planetary Science Letters, 481, 80–90. 10.1016/j.epsl.2017.10.023

[ggge22131-bib-0063] Ishizuka, O. , Kimura, J. I. , Li, Y. B. , Stern, R. J. , Reagan, M. K. , Taylor, R. N. , Ohara, Y. , Bloomer, S. H. , Ishii, T. , Hargrove, U. S. III , & Haraguchi, S. (2006). Early stages in the evolution of Izu–Bonin arc volcanism: New age, chemical, and isotopic constraints. Earth and Planetary Science Letters, 250(1‐2), 385–401. 10.1016/j.epsl.2006.08.007

[ggge22131-bib-0064] Ishizuka, O. , Tani, K. , Reagan, M. K. , Kanayama, K. , Umino, S. , Harigane, Y. , Sakamoto, I. , Miyajima, Y. , Yuasa, M. , & Dunkley, D. J. (2011). The timescales of subduction initiation and subsequent evolution of an oceanic island arc. Earth and Planetary Science Letters, 306(3‐4), 229–240. 10.1016/j.epsl.2011.04.006

[ggge22131-bib-0065] Ishizuka, O. , Taylor, R. N. , Yuasa, M. , & Ohara, Y. (2011). Making and breaking an island arc: A new perspective from the Oligocene Kyushu‐Palau arc, Philippine Sea. Geochemistry, Geophysics, Geosystems, 12, Q05005 10.1029/2010GC003440

[ggge22131-bib-0066] Jébrak, M. (1997). Hydrothermal breccias in vein‐type ore deposits: A review of mechanisms, morphology and size distribution. Ore Geology Reviews, 12(3), 111–134. 10.1016/S0169-1368(97)00009-7

[ggge22131-bib-0067] Jochum, K. P. , Scholz, D. , Stoll, B. , Weis, U. , Wilson, S. A. , Yang, Q. , Schwalb, A. , Börner, N. , Jacob, D. E. , & Andreae, M. O. (2012). Accurate trace element analysis of speleothems and biogenic calcium carbonates by LA‐ICP‐MS. Chemical Geology, 318, 31–44. 10.1016/j.chemgeo.2012.05.009

[ggge22131-bib-0068] John, C. M. , & Bowen, D. (2016). Community software for challenging isotope analysis: First applications of ‘Easotope’ to clumped isotopes. Rapid Communications in Mass Spectrometry, 30(21), 2285–2300. 10.1002/rcm.7720 27524507

[ggge22131-bib-0069] Kawahata, H. , Nohara, M. , Ishizuka, H. , Hasebe, S. , & Chiba, H. (2001). Sr isotope geochemistry and hydrothermal alteration of the Oman ophiolite. Journal of Geophysical Research ‐ Solid Earth, 106(B6), 11,083–11,099. 10.1029/2000JB900456

[ggge22131-bib-0070] Kele, S. , Breitenbach, S. F. , Capezzuoli, E. , Meckler, A. N. , Ziegler, M. , Millan, I. M. , Kluge, T. , Deák, J. , Hanselmann, K. , John, C. M. , Yan, H. , Liu, Z. , & Bernasconi, S. M. (2015). Temperature dependence of oxygen‐ and clumped isotope fractionation in carbonates: A study of travertines and tufas in the 6–95 °C temperature range. Geochimica et Cosmochimica Acta, 168, 172–192. 10.1016/j.gca.2015.06.032

[ggge22131-bib-0071] Kim, S. T. , & O'Neil, J. R. (1997). Equilibrium and nonequilibrium oxygen isotope effects in synthetic carbonates. Geochimica et Cosmochimica Acta, 61(16), 3461–3475. 10.1016/S0016-7037(97)00169-5

[ggge22131-bib-0072] Knight, C. L. , Williamson, M. A. , Bodnar, R. J. , & Russell, P. E. (1989). Raman spectroscopy of zeolites: Characterization of natural zeolites with the laser Raman microprobe. Microbeam Analysis–1989, 571–573.

[ggge22131-bib-0073] Koschinsky, A. (2014). Hydrothermal vent fluids (seafloor) In Harff J., Meschede M., Petersen S., & Thiede J. (Eds.), Encyclopedia of marine geosciences, (pp. 1–8). Dordrecht: Springer 10.1007/978-94-007-6644-0

[ggge22131-bib-0074] Kurz, W. , Micheuz, P. , Christeson, G. L. , Reagan, M. , Shervais, J. W. , Kutterolf, S. , Robertson, A. , Krenn, K. , Michibayashi, K. , & Quandt, D. (2019). Postmagmatic tectonic evolution of the outer Izu‐Bonin forearc revealed by sediment basin structure and vein microstructure analysis: Implications for a 15 Ma Hiatus between pacific plate subduction initiation and forearc extension. Geochemistry, Geophysics, Geosystems, 20, 5867–5895. 10.1029/2019GC008329 PMC7004124 32055237

[ggge22131-bib-0075] Lawrence, M. G. , Jupiter, S. D. , & Kamber, B. S. (2006). Aquatic geochemistry of the rare earth elements and yttrium in the Pioneer River catchment, Australia. Marine and Freshwater Research, 57(7), 725–736. 10.1071/MF05229

[ggge22131-bib-0076] Le Bas, M. J. (2000). IUGS reclassification of the high‐Mg and picritic volcanic rocks. Journal of Petrology, 41(10), 1467–1470. 10.1093/petrology/41.10.1467

[ggge22131-bib-0077] Lehner, B. L. (1991). Neptunian dykes along a drowned carbonate platform margin: an indication for recurrent extensional tectonic activity? Terra Nova, 3(6), 593–602. 10.1111/j.1365-3121.1991.tb00201.x

[ggge22131-bib-0078] McArthur, J. M. , Howarth, R. J. , & Bailey, T. R. (2001). Strontium isotope stratigraphy: LOWESS version 3: best fit to the marine Sr‐isotope curve for 0‐509 Ma and accompanying look‐up table for deriving numerical age. The Journal of Geology, 109(2), 155–170. 10.1086/319243

[ggge22131-bib-0079] McArthur, J. M. , Howarth, R. J. , & Shields, G. A. (2012). Strontium isotope stratigraphy. The geologic time scale, 1, 127–144.

[ggge22131-bib-0080] McBride, M. B. (1979). Chemisorption and Precipitation of Mn^2+^ at CaCO_3_ Surfaces. Soil Science Society of America Journal, 43(4), 693–698. 10.2136/sssaj1979.03615995004300040013x

[ggge22131-bib-0081] McCrea, J. M. (1950). On the isotopic chemistry of carbonates and a paleotemperature scale. The Journal of Chemical Physics, 18(6), 849–857. 10.1063/1.1747785

[ggge22131-bib-0082] McDonough, W. F. , & Sun, S. S. (1995). The composition of the Earth. Chemical Geology, 120(3‐4), 223–253. 10.1016/0009-2541(94)00140-4

[ggge22131-bib-0083] McLennan, S. M. (1989). Rare earth elements in sedimentary rocks: Influence of provenance and sedimentary processes. Geochemistry and Mineralogy of Rare Earth Elements, Reviews in Mineralogy, 21, 169–200.

[ggge22131-bib-0084] Means, W. D. , & Li, T. (2001). A laboratory simulation of fibrous veins: Some first observations. Journal of Structural Geology, 23(6‐7), 857–863. 10.1016/S0191-8141(00)00158-9

[ggge22131-bib-0085] Meckler, A. N. , Ziegler, M. , Millán, M. I. , Breitenbach, S. F. M. , & Bernasconi, S. M. (2014). Long‐term performance of the Kiel carbonate device with a new correction scheme for clumped isotope measurements. Rapid Communications in Mass Spectrometry, 28(15), 1705–1715. 10.1002/rcm.6949 24975251

[ggge22131-bib-0086] Meng, Q. , Hooker, J. N. , & Cartwright, J. (2019). Progressive accretion of antitaxial crystal fibres: Implications for the kinematics and dynamics of vein dilation. Journal of Structural Geology. 10.1016/j.jsg.2019.05.006

[ggge22131-bib-0087] Menzies, M. , & Seyfried, W. E. Jr. (1979). Basalt‐seawater interaction: Trace element and strontium isotopic variations in experimentally altered glassy basalt. Earth and Planetary Science Letters, 44(3), 463–472. 10.1016/0012-821X(79)90084-0

[ggge22131-bib-0088] Meyer, H. J. (1984). The influence of impurities on the growth rate of calcite. Journal of Crystal Growth, 66(3), 639–646. 10.1016/0022-0248(84)90164-7

[ggge22131-bib-0089] Möller, P. (2002). Rare earth elements and yttrium in geothermal fluids. Water Science and Technology Library, 40, 97–125.

[ggge22131-bib-0090] Muehlenbachs, K. , & Clayton, R. N. (1976). Oxygen isotope composition of the oceanic crust and its bearing on seawater. Journal of Geophysical Research, 81(23), 4365–4369. 10.1029/JB081i023p04365

[ggge22131-bib-0091] Müller, I. A. , Fernandez, A. , Radke, J. , Van Dijk, J. , Bowen, D. , Schwieters, J. , & Bernasconi, S. M. (2017). Carbonate clumped isotope analyses with the long‐integration dual‐inlet (LIDI) workflow: Scratching at the lower sample weight boundaries. Rapid Communications in Mass Spectrometry, 31(12), 1057–1066. 10.1002/rcm.7878 28402589

[ggge22131-bib-0092] Nollet, S. , Urai, J. L. , Bons, P. D. , & Hilgers, C. (2005). Numerical simulations of polycrystal growth in veins. Journal of Structural Geology, 27(2), 217–230. 10.1016/j.jsg.2004.10.003

[ggge22131-bib-0093] Nozaki, Y. , Zhang, J. , & Amakawa, H. (1997). The fractionation between Y and Ho in the marine environment. Earth and Planetary Science Letters, 148(1‐2), 329–340. 10.1016/S0012-821X(97)00034-4

[ggge22131-bib-0094] Okino, K. , Kasuga, S. , & Ohara, Y. (1998). A new scenario of the Parece Vela Basin genesis. Marine Geophysical Researches, 20(1), 21–40. 10.1023/A:1004377422118

[ggge22131-bib-0095] Ortoleva, P. , Merino, E. , Moore, C. , & Chadam, J. (1987). Geochemical self‐organization I; reaction‐transport feedbacks and modeling approach. American Journal of Science, 287(10), 979–1007. 10.2475/ajs.287.10.979

[ggge22131-bib-0096] Passey, B. H. , & Henkes, G. A. (2012). Carbonate clumped isotope bond reordering and geospeedometry. Earth and Planetary Science Letters, 351, 223–236. 10.1016/j.epsl.2012.07.021

[ggge22131-bib-0097] Pearson, W. C. , & Lister, C. R. B. (1973). Permeability measurements on a deep‐sea core. Journal of Geophysical Research, 78(32), 7786–7787. 10.1029/JB078i032p07786

[ggge22131-bib-0098] Petersen, S. V. , Defliese, W. F. , Saenger, C. , Daëron, M. , Huntington, K. W. , John, C. M. , Kelson, J. R. , Bernasconi, S. M. , Colman, A. S. , Kluge, T. , Olack, G. A. , Schauer, A. J. , Bajnai, D. , Bonifacie, M. , Breitenbach, S. F. M. , Fiebig, J. , Fernandez, A. B. , Henkes, G. A. , Hodell, D. , Katz, A. , Kele, S. , Lohmann, K. C. , Passey, B. H. , Peral, M. Y. , Petrizzo, D. A. , Rosenheim, B. E. , Tripati, A. , Venturelli, R. , Young, E. D. , & Winkelstern, I. Z. (2019). Effects of improved ^17^O correction on inter‐laboratory agreement in clumped isotope calibrations, estimates of mineral‐specific offsets, and temperature dependence of acid digestion fractionation. Geochemistry, Geophysics, Geosystems, 20(7), 3495–3519. 10.1029/2018GC008127

[ggge22131-bib-0099] Quandt, D. , Micheuz, P. , Kurz, W. , Bernasconi, S. M. , Hippler, D. , Krenn, K. , & Hauzenberger, C. (2019). Trace element concentrations and clumped isotope compositions of calcite veins pervading the Western Pacific Izu‐Bonin forearc and rear arc crust. PANGAEA.. 10.1594/PANGAEA.906261

[ggge22131-bib-0100] Quandt, D. , Micheuz, P. , Kurz, W. , Kluge, T. , Boch, R. , Hippler, D. , Krenn, K. , & Hauzenberger, C. A. (2019). Geochemistry of vein calcites hosted in the Troodos Pillow Lavas and their implications for the timing and physicochemical environment of fracturing, fluid circulation, and vein mineral growth. Geochemistry, Geophysics, Geosystems, 20, 5913–5938. 10.1029/2019GC008369 PMC7004035 32055238

[ggge22131-bib-0101] Quandt, D. , Micheuz, P. , Kurz, W. , & Krenn, K. (2018). Microtextures and fluid inclusions from vein minerals hosted in the Pillow Lavas of the Troodos supra‐subduction zone. Lithosphere, 10(4), 566–578. 10.1130/L696.1

[ggge22131-bib-0102] Ramsay, J. G. (1980). The crack‐seal mechanism of rock deformation. Nature, 284(5752), 135–139. 10.1038/284135a0

[ggge22131-bib-0103] Ramsay, J. G. , & Huber, M. I. (1983). The techniques of modern structural geology: Strain analysis (Vol. 1), (pp. 1–307). London, UK: Academic Press.

[ggge22131-bib-0104] Reagan, M. K. , Heaton, D. E. , Schmitz, M. D. , Pearce, J. A. , Shervais, J. W. , & Koppers, A. A. (2019). Forearc ages reveal extensive short‐lived and rapid seafloor spreading following subduction initiation. Earth and Planetary Science Letters, 506, 520–529. 10.1016/j.epsl.2018.11.020

[ggge22131-bib-0105] Reagan, M. K. , Ishizuka, O. , Stern, R. J. , Kelley, K. A. , Ohara, Y. , Blichert‐Toft, J. , Bloomer, S. H. , Cash, J. , Fryer, P. , Hanan, B. B. , Hickey‐Vargas, R. , Ishii, T. , Kimura, J.‐I. , Peate, D. W. , Rowe, M. C. , & Woods, M. (2010). Fore‐arc basalts and subduction initiation in the Izu‐Bonin‐Mariana system. Geochemistry, Geophysics, Geosystems, 11, Q03X12 10.1029/2009GC002871

[ggge22131-bib-0106] Reagan, M. K. , McClelland, W. C. , Girard, G. , Goff, K. R. , Peate, D. W. , Ohara, Y. , & Stern, R. J. (2013). The geology of the southern Mariana fore‐arc crust: Implications for the scale of Eocene volcanism in the western Pacific. Earth and Planetary Science Letters, 380, 41–51. 10.1016/j.epsl.2013.08.013

[ggge22131-bib-0107] Reagan, M. K. , Pearce, J. A. , Petronotis, K. , Almeev, R. R. , Avery, A. J. , Carvallo, C. , Chapman, T. , Christeson, G. L. , Ferré, E. C. , Godard, M. , Heaton, D. E. , Kirchenbaur, M. , Kurz, W. , Kutterolf, S. , Li, H. , Li, Y. , Michibayashi, K. , Morgan, S. , Nelson, W. R. , Prytulak, J. , Python, M. , Robertson, A. H. F. , Ryan, J. G. , Sager, W. W. , Sakuyama, T. , Shervais, J. W. , Shimizu, K. , & Whattam, S. A. (2017). Subduction initiation and ophiolite crust: New insights from IODP drilling. International Geology Review, 59(11), 1439–1450. 10.1080/00206814.2016.1276482

[ggge22131-bib-0108] Reagan, M. K. , Pearce, J. A. , Petronotis, K. , & the Expedition 352 Scientists (2015). Izu‐Bonin‐Mariana fore arc. Proceedings of the International Ocean Discovery Program, Expedition 352. College Station, TX (International Ocean Discovery Program). 10.14379/iodp.proc.352.2015

[ggge22131-bib-0109] Reeder, R. J. , Fagioli, R. O. , & Meyers, W. J. (1990). Oscillatory zoning of Mn in solution‐grown calcite crystals. Earth‐Science Reviews, 29(1‐4), 39–46. 10.1016/0012-8252(0)90026-R

[ggge22131-bib-0110] Richardson, S. H. , Hart, S. R. , & Staudigel, H. (1980). Vein mineral ages of old oceanic crust. Journal of Geophysical Research ‐ Solid Earth, 85(B12), 7195–7200. 10.1029/JB085iB12p07195

[ggge22131-bib-0111] Roberts, N. M. , & Walker, R. J. (2016). U‐Pb geochronology of calcite‐mineralized faults: Absolute timing of rift‐related fault events on the northeast Atlantic margin. Geology, 44(7), 531–534. 10.1130/G37868.1

[ggge22131-bib-0112] Robertson, A. H. , Kutterolf, S. , Avery, A. , Baxter, A. T. , Petronotis, K. , Acton, G. D. , Carvallo, C. , & Schindlbeck, J. C. (2018). Depositional setting, provenance, and tectonic‐volcanic setting of Eocene–Recent deep‐sea sediments of the oceanic Izu–Bonin forearc, northwest Pacific (IODP Expedition 352). International Geology Review, 60(15), 1816–1854. 10.1080/00206814.2017.1393634

[ggge22131-bib-0113] Sample, J. C. , Torres, M. E. , Fisher, A. , Hong, W. L. , Destrigneville, C. , Defliese, W. F. , & Tripati, A. E. (2017). Geochemical constraints on the temperature and timing of carbonate formation and lithification in the Nankai Trough, NanTroSEIZE transect. Geochimica et Cosmochimica Acta, 198, 92–114. 10.1016/j.gca.2016.10.013

[ggge22131-bib-0114] Schier, K. , Bau, M. , Münker, C. , Beukes, N. , & Viehmann, S. (2018). Trace element and Nd isotope composition of shallow seawater prior to the Great Oxidation Event: Evidence from stromatolitic bioherms in the Paleoproterozoic Rooinekke and Nelani Formations, South Africa. Precambrian Research, 315, 92–102. 10.1016/j.precamres.2018.07.014

[ggge22131-bib-0115] Schmid, T. W. , & Bernasconi, S. M. (2010). An automated method for “clumped‐isotope” measurements on small carbonate samples. Rapid Communications in Mass Spectrometry, 24(14), 1955–1963. 10.1002/rcm.4598 20552704

[ggge22131-bib-0116] Schroeder, T. , Bach, W. , Jöns, N. , Jöns, S. , Monien, P. , & Klügel, A. (2015). Fluid circulation and carbonate vein precipitation in the footwall of an oceanic core complex, Ocean Drilling Program Site 175, Mid‐Atlantic Ridge. Geochemistry, Geophysics, Geosystems, 16, 3716–3732. 10.1002/2015GC006041

[ggge22131-bib-0117] Sdrolias, M. , Roest, W. R. , & Müller, R. D. (2004). An expression of Philippine Sea plate rotation: The Parece Vela and Shikoku basins. Tectonophysics, 394(1‐2), 69–86. 10.1016/j.tecto.2004.07.061

[ggge22131-bib-0118] Shervais, J. W. , Reagan, M. , Haugen, E. , Almeev, R. R. , Pearce, J. A. , Prytulak, J. , Ryan, J. G. , Whattam, S. A. , Godard, M. , Chapman, T. , Li, H. , Kurz, W. , Nelson, W. R. , Heaton, D. , Kirchenbaur, M. , Shimizu, K. , Sakuyama, T. , Li, Y. , & Vetter, S. K. (2019). Magmatic response to subduction initiation: Part 1. Fore‐arc basalts of the Izu‐Bonin arc from IODP Expedition 352. Geochemistry, Geophysics, Geosystems, 20(1), 314–338. 10.1029/2018GC007731 PMC6392113 30853858

[ggge22131-bib-0119] Shore, M. , & Fowler, A. D. (1996). Oscillatory zoning in minerals; a common phenomenon. The Canadian Mineralogist, 34(6), 1111–1126.

[ggge22131-bib-0120] Stammeier, J. A. , Hippler, D. , Nebel, O. , Leis, A. , Grengg, C. , Mittermayr, F. , Kasemann, S. A. , & Dietzel, M. (2018). Radiogenic Sr and Stable C and O isotopes across Precambrian‐Cambrian transition in marine carbonatic phosphorites of Malyi Karatau (Kazakhstan)—Implications for paleo‐environmental change. Geochemistry, Geophysics, Geosystems, 20, 3–23. 10.1029/2018GC007767

[ggge22131-bib-0121] Staudigel, H. , Davies, G. R. , Hart, S. R. , Marchant, K. M. , & Smith, B. M. (1995). Large scale isotopic Sr, Nd and O isotopic anatomy of altered oceanic crust: DSDP/ODP Sites 417/418. Earth and Planetary Science Letters, 130(1‐4), 169–185. 10.1016/0012-821X(94)00263-X

[ggge22131-bib-0122] Staudigel, H. , & Hart, S. R. (1985). Dating of ocean crust hydrothermal alteration‐strontium isotope ratios from Hole‐504B carbonates and a reinterpretation of Sr isotope data from Deep‐Sea Drilling Project Sites 105, 332, 417, and 418. Initial Reports of the Deep Sea Drilling Project, 83(APR), 297–303.

[ggge22131-bib-0123] Staudigel, H. , Muehlenbachs, K. , Richardson, S. H. , & Hart, S. R. (1981). Agents of low temperature ocean crust alteration. Contributions to Mineralogy and Petrology, 77(2), 150–157. 10.1007/BF00636518

[ggge22131-bib-0124] Stern, R. J. , & Bloomer, S. H. (1992). Subduction zone infancy: Examples from the Eocene Izu‐Bonin‐Mariana and Jurassic California arcs. Geological Society of America Bulletin, 104(12), 1621–1636. 10.1130/0016-7606(1992)104<1621:SZIEFT>2.3.CO;2

[ggge22131-bib-0125] Stolper, D. A. , & Eiler, J. M. (2015). The kinetics of solid‐state isotope‐exchange reactions for clumped isotopes: A study of inorganic calcites and apatites from natural and experimental samples. American Journal of Science, 315(5), 363–411.

[ggge22131-bib-0126] Sverjensky, D. A. (1984). Europium redox equilibria in aqueous solution. Earth and Planetary Science Letters, 67(1), 70–78. 10.1016/0012-821X(84)90039-6

[ggge22131-bib-0127] Taber, S. (1916). The origin of veins of the asbestiform minerals. Proceedings of the National Academy of Sciences of the United States of America, 2(12), 659–664. 16586653 10.1073/pnas.2.12.659 PMC1091131

[ggge22131-bib-0128] Tartarotti, P. , & Pasquaré, F. A. (2003). Basaltic breccias in the upper oceanic crust, Hole 504b (Costa Rica Rift, Pacific Ocean). Ofioliti, 28(1), 59–67.

[ggge22131-bib-0129] Tostevin, R. , Shields, G. A. , Tarbuck, G. M. , He, T. , Clarkson, M. O. , & Wood, R. A. (2016). Effective use of cerium anomalies as a redox proxy in carbonate‐dominated marine settings. Chemical Geology, 438, 146–162. 10.1016/j.chemgeo.2016.06.027

[ggge22131-bib-0130] Urey, H. C. (1947). The thermodynamic properties of isotopic substances. Journal of the Chemical Society (Resumed), 562–581. 10.1039/JR9470000562 20249764

[ggge22131-bib-0131] Wang, Y. , & Merino, E. (1992). Dynamic model of oscillatory zoning of trace elements in calcite: Double layer, inhibition, and self‐organization. Geochimica et Cosmochimica 1341 Acta, 56(2), 587–596. 10.1016/0016-7037(92)90083-U

[ggge22131-bib-0132] Williams, P. F. , & Urai, J. L. (1989). Curved vein fibres: An alternative explanation. Tectonophysics, 158(1‐4), 311–333. 10.1016/0040-1951(89)90330-2

[ggge22131-bib-0133] Wiltschko, D. V. , & Morse, J. W. (2001). Crystallization pressure versus “crack seal” as the mechanism for banded veins. Geology, 29(1), 79–82. 10.1130/0091-7613(2001)029<0079:CPVCSA>2.0.CO;2

[ggge22131-bib-0134] Yogodzinski, G. M. , Bizimis, M. , Hickey‐Vargas, R. , McCarthy, A. , Hocking, B. D. , Savov, I. P. , Ishizuka, O. , & Arculus, R. (2018). Implications of Eocene‐age Philippine Sea and forearc basalts for initiation and early history of the Izu‐Bonin‐Mariana arc. Geochimica et Cosmochimica Acta, 228, 136–156. 10.1016/j.gca.2018.02.047

[ggge22131-bib-0135] Zachos, J. , Pagani, M. , Sloan, L. , Thomas, E. , & Billups, K. (2001). Trends, rhythms, and aberrations in global climate 65 Ma to present. Science, 292(5517), 686‐693. DOI: 10.1126/science.1059412. DOI: 10.1126/science.1059412 11326091

[ggge22131-bib-0136] Zhang, J. , & Nozaki, Y. (1996). Rare earth elements and yttrium in seawater: ICP‐MS determinations in the East Caroline, Coral Sea, and South Fiji basins of the western South Pacific Ocean. Geochimica et Cosmochimica Acta, 60(23), 4631–4644. 10.1016/S0016-7037(96)00276-1

